# Assessing the impact of technological change on similar occupations: Implications for employment alternatives

**DOI:** 10.1371/journal.pone.0291428

**Published:** 2023-09-18

**Authors:** Karine Torosyan, Sicheng Wang, Elizabeth A. Mack, Jenna A. Van Fossen, Nathan Baker

**Affiliations:** 1 Department of Geography, Environment and Spatial Sciences, Michigan State University, East Lansing, Michigan, United States of America; 2 International School of Economics in Tbilisi (ISET), Tbilisi State University, Tbilisi, Georgia; 3 Department of Geography, University of South Carolina, Columbia, South Carolina, United States of America; 4 Department of Psychology, Clemson University, Clemson, South Carolina, United States of America; 5 Department of Psychology, Michigan State University, East Lansing, Michigan, United States of America; Estrella Mountain College, UNITED STATES

## Abstract

**Background:**

The fast-changing labor market highlights the need for an in-depth understanding of occupational mobility impacted by technological change. However, we lack a multidimensional classification scheme that considers similarities of occupations comprehensively, which prevents us from predicting employment trends and mobility across occupations. This study fills the gap by examining employment trends based on similarities between occupations.

**Method:**

We first demonstrated a new method that clusters 756 occupation titles based on knowledge, skills, abilities, education, experience, training, activities, values, and interests. We used the Principal Component Analysis to categorize occupations in the Standard Occupational Classification, which is grouped into a four-level hierarchy. Then, we paired the occupation clusters with the occupational employment projections provided by the U.S. Bureau of Labor Statistics. We analyzed how employment would change and what factors affect the employment changes within occupation groups. Particularly, we specified factors related to technological changes.

**Results:**

The results reveal that technological change accounts for significant job losses in some clusters. This poses occupational mobility challenges for workers in these jobs at present. Job losses for nearly 60% of current employment will occur in low-skill, low-wage occupational groups. Meanwhile, many mid-skilled and highly skilled jobs are projected to grow in the next ten years.

**Conclusion:**

Our results demonstrate the utility of our occupational classification scheme. Furthermore, it suggests a critical need for skills upgrading and workforce development for workers in declining jobs. Special attention should be paid to vulnerable workers, such as older individuals and minorities.

## Introduction

Technological change is a disruptive force on labor markets, and studies find that jobs are becoming increasingly polarized into low and high-skilled jobs [1–4]. Recent technological innovations related to robotics and artificial intelligence are anticipated to have particularly disruptive impacts on the workforce [5]. By 2025, projections indicate that these changes will create 97 million new jobs and destroy 85 million current jobs globally [6]. A concern about this recent wave of new technologies is that they will begin to replace high-skill, high-wage jobs [7, 8]; thereby placing previously stable work in a precarious position.

Two economic theories help explain this workforce polarization. The theory of skills-biased technological change (SBTC) predicts that the demand for highly skilled workers, and the wages they earn, rises during periods of technological change [1]. Based on this theory, technology complements highly skilled jobs and substitutes for jobs with low and medium skills, leading to a rise in income inequality [3]. Instead, the theory of routine-biased technological change (RBTC) [9] offers a more nuanced perspective on how advances in computing technologies replace human labor in routine tasks, which can be decomposed into step-by-step procedures or rules. Thus, the RBTC framework helps explain the disappearance of the “middle” in both manual and cognitive-related jobs that are replaceable by machines due to the predictable, routine aspects of work [2]. On the other hand, non-routine jobs, either highly-skilled or unskilled, are likely to increase as complements to technologies [2]. Therefore, the projection of job demand requires a comprehensive assessment of all occupational attributes beyond skills, including knowledge, experience, training, activities, interests, and values.

One of the important aspects of technological change in the labor market to consider is its impact on occupational mobility or the ability of workers to move between jobs. This is important to evaluate for workers who are displaced by technology. Based on the theory of RBTC, one would expect workers employed in non-routine jobs with a high cognitive task content to be more mobile than workers in both routine manual and routine cognitive work. Cortes [10] evaluated occupational mobility in the US between 1976 and 2007 and categorized occupations into the three groups proposed by Autor et al. [9]. They find routine workers experienced a fall in their wage premiums while people who moved out of routine jobs were more likely to experience wage growth. Maczulskij [11] examined the occupational mobility of mid-skilled routine workers in Finland and found differences between routine cognitive workers and routine manual workers. In particular, they find that routine cognitive workers can move to abstract tasks while routine manual workers tend to move to service tasks. Goos et al. [12] examine the reemployment prospects of workers after plant closure in Belgium using an RBTC framework. They find evidence in support of this theory. The reemployment prospects for workers performing non-routine tasks and with digital skills were better than for workers accustomed to performing routine tasks. That said, few studies have examined the impact of technological change on occupational mobility, particularly in a U.S. context, and more work needs to be done in this area [11, 13].

Analyses of occupational mobility classify occupations into groups based on their similarity in characteristics (e.g., wages, skills, tasks) [12, 14–24]. The logic behind the creation of these groups is that movement between similar jobs *within* groups is easier than movement *between* groups where jobs are more dissimilar. Thus, the availability of more alternatives within an occupational cluster enhances the number of similar employment alternatives for workers. A common approach of occupational mobility studies is to cluster occupations with a focus on one dimension. For example, some studies categorize occupations based on skills [25], tasks [26], or work values and interests [27–29]. In particular, principal components analysis (PCA) has been used to reduce very large numbers of skills or tasks to assist in classifying jobs [17, 25, 30]. Considering different occupational characteristics, although there has been some dispute over the distinction between tasks and skills, Yamaguchi [31] delineated between tasks and skills and proposed that more observable tasks are indicative of particular skills. It should be noted that there are some existing occupational classification systems, such as the International Standard Classification of Occupations (ISCO) and Standard Occupational Classification (SOC). The ISCO system is not recommended for US-based analysis because “it is not flexible enough for US needs” [32]. The SOC system, established by the Office of Management and Budget, is for use in the Federal statistical system in the US [33]. However, the principle of the SOC classifications is based on “work performed” and “the highest level of skills” required in each occupation [32, 33], which does not thoroughly reflect similarities regarding occupational attributes, human capital requirements, and activities. Therefore, SOC classifications may not be sufficient for occupational mobility studies.

The Occupational Classification Network (O*NET) data, which indexes detailed occupational attributes for US occupations, has been widely used in grouping occupations and examining the job characteristics’ relatedness [33–43]. Hundreds of attributes, termed as descriptors, are divided into different dimensions, such as knowledge, skills, abilities, education, experience, training, interests, work values, work styles, tasks, technology skills & tools, work activities, and work contents. Each descriptor is rated with a scale. Therefore, analysts can examine the relationships between descriptors based on the values. The data is provided to “help people find the training and jobs they need, and employers the skilled workers” [34]. However, it is difficult to identify alternatives for a worker in a specific occupation because O*NET does not group occupations based on the values of its descriptors. Some studies, such as Feser [35], Koo [36], Nolan et al. [37], Slaper [38] and Burrus et al. [39], used PCA clustering with the Ward algorithm to determine the similarity based on some occupational descriptors in O*NET. Feser [35], Koo [36], and Nolan et al. [37] all created clusters based on the “knowledge” descriptors. Slaper [38] used “knowledge,” “skills,” and “training” in clustering. Burrus et al. [39] used “knowledge,” “skill,” “ability,” and “work style.” Manzella et al. [40] used structured and unstructured factor analysis to group occupations based on “task” descriptors. Yu et al. [41] used PCA with a hierarchical cluster analysis to group occupations at two levels. Their analysis was based on descriptors of “knowledge,” “skills,” “abilities,” and “training,” but the higher-level clusters considered only job training requirements. Waters and Shutters [42] and their earlier work [43] created a skills network based on values of six categories of descriptors in O*NET (knowledge, skills, interests, work activities, work styles, and work values) and employment in an occupation in a metropolitan area. Alabdulkareem et al. [44] and Farinha et al. [45] used revealed comparative advantage (RCA, also called a location quotient) to determine whether an O*NET attribute is present or absent in an occupation. RCA helped calculated network-based similarity by examining the relatedness and complementarity of occupational attributes in O*NET. The former study [44] used the importance scale for 161 descriptors of “knowledge,” “skills,” and “abilities,” and the latter [45] used descriptors of “Intermediate Work Activities” (IWA), a sub-category of work activities.

This study develops a nested occupational classification scheme based on the theory of human capital. In Becker’s [46] theory of human capital, human capital is defined with multiple dimensions, namely knowledge, skills, abilities, health, and values, with education and training identified as the most important ways to invest in human capital. And indeed, there is ample empirical evidence demonstrating the importance of these factors in determining labor market outcomes, including job placement and mobility [47].

Conceptualizing similarity in terms of skills, tasks, abilities, training, and related information may indicate whether a worker can enter a job. However, it is also valuable to incorporate information on values and interests to get a sense of worker performance and satisfaction in a new job. This is particularly the case given the more recent focus of research on meaningful aspects of work, as people are beginning to view jobs as more than a means of making an income [48, 49]. Interests and values predict long-term performance and turnover in a given job [50–52]. A recent meta-analysis conducted a quantitative review of interests and career choice over 100 years, suggesting that measured interests attain a hit rate of 51% for predicting career choice [53]. The aforementioned studies, such as Shutters and Waters [43] and Waters and Shutters [42] have already included interest elements in occupational clustering. Values, meanwhile, reflect beliefs that motivate evaluations of what environmental characteristics and actions are most preferable [54, 55], influencing how well an employee fits within a given role and, thus, their likelihood of turnover [56]. Determinations about the work values that a given occupation satisfies are based on the theory of work adjustment [57], which views values (e.g., achievement, independence) as factors that influence what elements of a given job a worker finds desirable. Similarly, vocational interests reflect preferences for certain types of environments and activities [58]. Holland’s [50] RIASEC is the most popular model of vocational interests, proposing that people differ along six stable work interests: realistic, investigative, artistic, social, enterprising, and conventional. Holland postulates that people select careers that fit their personality orientations, as closer alignment between personality and work environment should lead to better job experiences. Unlike knowledge and skills which a worker must acquire when transitioning from one occupation to another, integrating values and interests into occupational clustering can provide job seekers with a sense that the jobs may be personally satisfying, which could enhance their satisfaction, performance, and long-term retention in alternative occupations.

In conclusion, the current literature lacks studies that project employment trends and mobility across occupations based on similarities in multiple factors. Moreover, although prior studies have explored occupation clustering in various approaches, we lack a comprehensive classification scheme for predicting employment trends and occupational mobility driven by technological change. Our paper builds on prior work focused on occupational clustering by creating a new multidimensional classification scheme of jobs. We move beyond prior work focused on occupational clustering to consider the impact of technological change on the size of occupational clusters. This a priori step is important because technological change may eliminate several jobs within occupational clusters, limiting the number of occupational alternatives for workers and their ability to find jobs. Based on human capital theory, we cluster over 700 occupational titles across their knowledge, skills and abilities (KSA), human capital requirements (education, experience, and training (EET)) and work activities, values and interests (AVI). The classification method considers numerous dimensions of potential similarity across occupations, including skills, tasks, human capital-related occupational characteristics (e.g., experience and educational attainment), work values, and interests. Some dimensions, particularly work interests, are important to incorporate in occupational classifications because they have meaningful implications for occupational transitions [53, 59, 60]. The four-level nested hierarchical occupation classifications provide for a finer level of classification. More importantly, we pair this output with information about the drivers of growth or decline for particular occupations from the Bureau of Labor Statistics (BLS) to answer two questions. One, which occupations will grow or decline because of technological change? Two, to what extent does technological change eliminate occupations within clusters? This final question is important for assessing future employment alternatives for workers displaced by technological change. If entire clusters are displaced by technological change, the occupational mobility of displaced workers could be severely limited.

By examining the employment projections for occupations clustered in our analysis, we reveal that job losses of nearly 60% of current employment will occur in low-skill, low-wage occupational groups. On the other hand, primary job gains will be made in high-wage high-educational attainment jobs, which require a Master’s degree or higher. Our analysis also highlights future growth in high-wage, high-educational attainment occupations as well as occupations in the “middle” portion of the spectrum. These “middle” occupations include various types of technician, programming, detective and investigative work, financial examiners and police work. While work in these occupations could be somewhat routine, they also require a human reasoning element that cannot be replaced by machines right now. Much of this job growth is related to Internet-related technologies and the Internet of Things (IoT). This growth in mid-wage jobs suggests that routine-biased technological change (RBTC) is not projected to occur in the short run (i.e., 2029). Moving forward, the wages of these jobs should be tracked to understand long-term trends in wages.

From a policy perspective, the disappearance of work at the lower end of the spectrum means finding a way of retraining workers so they are better equipped to cope with labor market changes wrought by technological change. For a long time, employers in the US have not done an effective job of providing on-the-job training [61, 62]. The nested occupational clustering based on O*NET data provides a foundation for developing worker training and certificate programs by identifying knowledge, skills, and educational gaps between declining and growing occupations. Moreover, the growing inequality in educational opportunities throughout childhood and young adulthood has been exacerbated by technological advances [63, 64]. Our analyses of wage patterns and employment trends within occupational clusters shed light on policymaking that aims to mitigate technological gaps in educational opportunities. Finally, integrating information about values and interests satisfied in occupations into groupings of similarity can also provide job seekers with a sense of the occupations that may be personally satisfying, which could increase their satisfaction and long-term retention in alternative occupations.

## Materials and methods

### Data

To understand the impact of technological change on jobs within occupation clusters, we use information from three data sources. O*NET database (version 25.0) provides hundreds of detailed attributes of occupational characteristics, requirements, and activities for US occupations, described as job descriptors [65]. For every indexed occupation, the descriptors are categorized into a variety of categories, including knowledge, skills, abilities (KSA), education, experience, training (EET), work activities, values, and interests (AVI), as well as tasks, and technology skills and tools [66]. Every category is indexed in a separate data file. For example, the “knowledge” file includes descriptors such as “Administration and Management,” “Communications and Media,” and “Chemistry.” For each occupation, O*NET associates each descriptor with a scale with a numeric range, such as importance, level, relevance, frequency, occupational interest, extent of the activity, and content. Appendices A-C in S2 Appendix list the KSA, EET, and AVI elements from the O*NET database used in our analysis for 220 descriptors. O*NET collects data for over 900 occupations categorized based on the Standard Occupational Classification System (SOC). We work with occupations based on 2018 SOC occupational codes (722 titles). In cases where data for 2018 SOC occupations were not collected (149 titles), we aggregate data from more detailed classification codes (2018 SOC occupational descriptors are computed as the averages of descriptors for lower-level titles included in a given occupation). This produces a final list of 756 occupations to cluster.

After clustering the occupations, we examine occupational wage variation within clusters. We use May 2019 wage and employment estimates from the Occupational Employment Statistics (OES) program from the US Bureau of Labor Statistics (BLS) to examine wages within clusters. The OES survey reports estimated wages by occupation (following the 2010 SOC system), which includes straight-time gross pay and excludes any bonuses and nonwage benefits. An occupational mean annual wage estimate is calculated by summing the annual wages of all the employees in a given occupation and dividing the total wages by the number of employees in that occupation.

We also use data from the Employment Projections (EP) program of the BLS, which publishes 10-year projections of national employment by industry and occupation [67]. The projection is based on analyses of historical and present economic statistics for the labor force, aggregate economic growth, commodity final demand, input-output, industry output and employment, job openings, wage and salary, and factors affecting demand for occupations, including technological innovation, changes in business or production models, product or service replacement, organizational restructuring of work, changes to the size of business establishments, and offshore outsourcing [68]. The EP data used in this study provide projections for 2019–2029. In addition to the projected number of jobs by occupation, EP tables [69] contain a summary of the main factors behind projected changes in reported occupations. This enables us to understand why jobs are projected to grow or decline. A helpful feature of the explanations associated with the job growth or decline for each occupation is that the description outlines if the changes are due to changes in technology. We integrate this information with our clustering scheme to analyze technology-driven job dynamics within occupational groups.

### Methods

We use the O*NET data release version 25.0 (August 2020) [66] to extract the 220 occupational descriptors discussed in the data section. Many of these descriptors are highly correlated with each other: the pairwise correlation values range from -0.76 to 0.97. To reduce the dimensionality of the information in this database and to produce a set of orthogonal descriptors of occupations, we perform Principal Component Analysis (PCA) using the standardized versions of the original variables and retain the first 72 principal components. These 72 principal components account for more than 90% of the total variation in the data and are used to perform cluster analysis.

**Fig 1:**
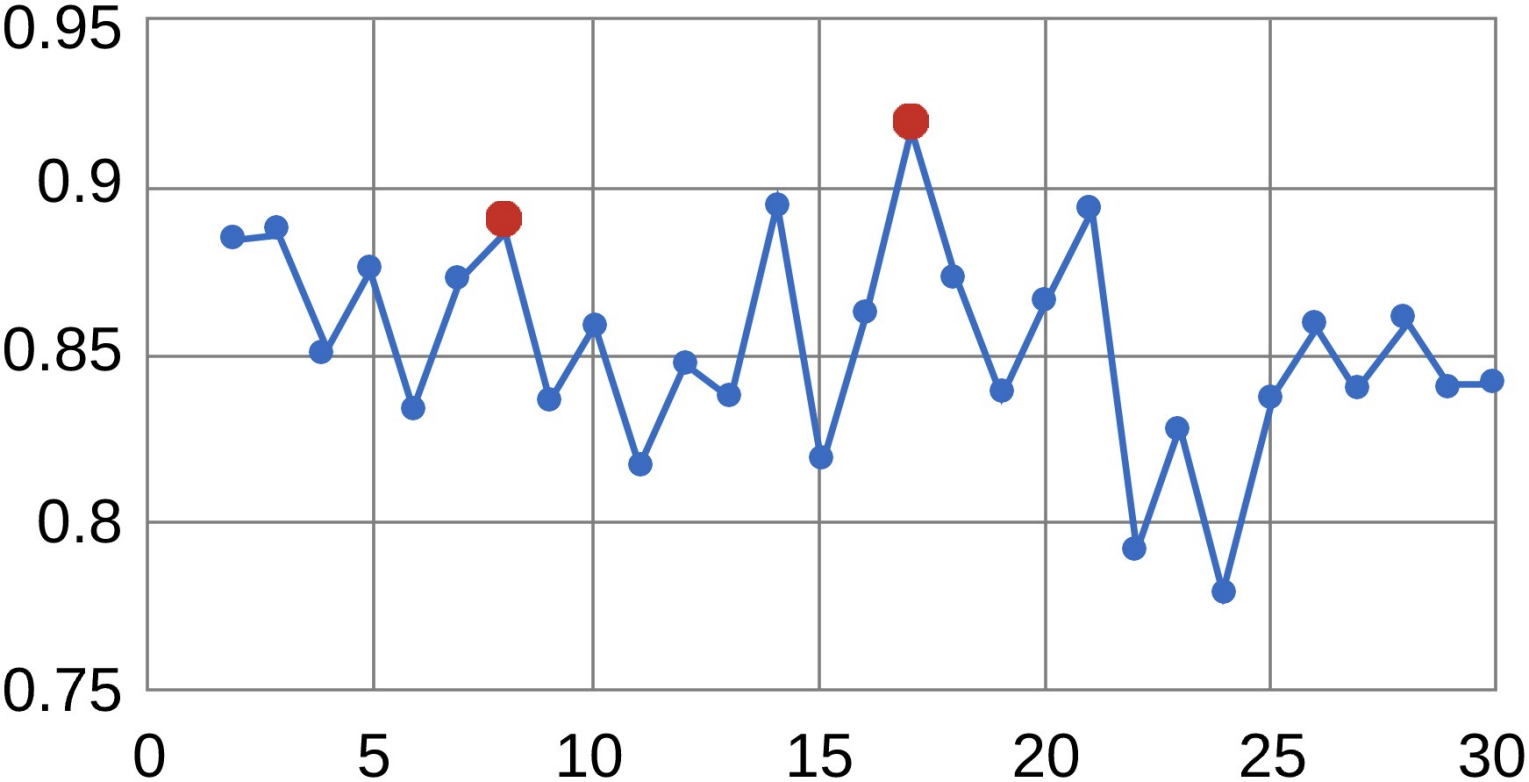
Duda-hart index and points of local maxima for 8 and 17-group splits (in red). **Sources**: Ward hierarchical clustering method based on 72 principal components derived from 220 occupational descriptors from O*NET.

To cluster occupations, we use the Ward agglomerative hierarchical clustering technique to create a hierarchy of occupations. This choice of method is driven by the following considerations: a) we aim to create a hierarchy of occupations–from more aggregate to less aggregate, and the use of a hierarchical method helps to achieve this goal (partitioning methods do not produce a clear hierarchy of clusters), b) we want to avoid creating clusters of heterogeneous size–a situation which is typical when using some hierarchical clustering methods, most notably single- and average-linkage methods, but less common with Ward’s method. The similarity between observations is determined based on a squared Euclidean distance-based measure. This selection of the distance measure is based on prior work, which warns against using other measures of distance with Ward’s clustering [70–73]. To determine the stopping points for different classification levels, we use the Duda and Hart [74] statistic, which pairs well with agglomerative clustering algorithms [75]. Large values of the Duda-Hart (DH) statistic indicate a distinct cluster structure for the given number of clusters, while small values indicate a less clearly defined cluster structure. Fig 1 graphs the DH statistic based on Ward’s hierarchical clustering of occupations. Points of local maxima indicate the most meaningful splits in the occupational hierarchy.

Based on the DH statistics, we select 2 points of local maxima as our stopping points: these are 8- and 17-group splits with the value of DH statistic of 0.89 and 0.92, respectively. Based on these considerations, we construct the following taxonomy of occupations:

- Level 1: 8 Occupational Clusters
- Level 2: 17 Occupational Groups

In the technical appendix (S1 Appendix), we extend the taxonomy of occupations to include a 40-group split (Level 3) and a 59-group split (Level 4), and the complete list of the taxonomy is presented in Appendix D in S2 Appendix.

Our clustering algorithm does not use wages to group occupations, so this is an external variable that we use to assess the quality of our clustering results. The rationale for doing so is that occupations with similar human capital requirements should pay similar wages (e.g., [76]). To assess the homogeneity in wages within clusters, we compute the sum of squares within groups (SSW):

$$SSW_{c}=\overset{N_{c}}{\underset{j=1}{\sum }}\overset{n_{j}}{\underset{i=1}{\sum }}{\left(w_{ij}-{\bar{w}}_{j}\right)}^{2}$$

where *w*_*ij*_ represents the annual mean wage for occupation *i* within Cluster *j*, ${\bar{w}}_{j}$ is the average wage within Cluster *j*, *n*_*j*_ is the size of Cluster *j*, and *N*_*c*_ = 8 and 17 is the number of clusters at a given classification level c = 1, 2. Next, we estimate the upper bound for the sum of squares within groups (SSW_U_) based on a random assignment of wages into the existing cluster structure. This scenario corresponds to the case when the clustering scheme does not explain any variation in wages. The lower bound for the sum of squares within groups (SSW_L_) is calculated by partitioning sorted wages into the existing cluster structure. This scenario corresponds to the maximum “fit” given the cluster structure and observed wage data. The following goodness-of-fit measure should reflect where the actual SSW falls between these two extremes:

$$Fit_{c}=\frac{SSW_{c,U}-SSW_{c}}{SSW_{c,U}-SSW_{c,L}}$$

Table 1 provides the resulting goodness-of-fit measure for our two levels of classification based on occupation-level wage data from the OES. At the most aggregate level of classification, 38% of the variation in wages is captured by our cluster structure. The fit improves as we move to a finer classification level, reaching 49% for the 17-group solution. Considering wages are not directly utilized in clustering, this level of fit based on wages suggests that our cluster solutions lead to compact groupings of occupations, especially at a finer level of classifications.

**Table 1:** Wage sums of squares within clusters and goodness of fit.

Level of clustering	SSW			Goodness of Fit
	Actual	Lower bound	Upper bound	
c = 1 (8 clusters)	552,280	13,256	883,236	0.38
c = 2 (17 groups)	448,671	12,946	875,489	0.49

To evaluate employment patterns and trends within clusters, we use EP data from the Bureau of Labor Statistics. The EP data estimate the employment trends by occupation in the next decade [67]. The data used in this study were projected employment trends for 2019–2029. There were 162.8 million people employed in 2019 (in occupations covered in BLS projections), and the projected number employed in 2029 is 168.9 million. This is equivalent to a 3.7% growth rate over the entire decade, a very conservative projection. For comparison, the projected growth in jobs for the 2009–2019 period was as high as 13.7% (based on the BLS archive), and the actual growth for the same period was around 14.9% [77]. So, the overall expansion in the employment base in the next decade is projected to be modest. Taking the 3.7% growth rate as a baseline scenario, we identify slower and faster-growing occupations and calculate the negative or positive gaps in the number of jobs within each occupation compared to this baseline scenario. We then aggregate these gaps by occupational groupings to compute the total number of jobs that will be lost and gained within each grouping in the coming decade, compared to the baseline.

For most occupations with sizable changes in employment, the BLS reports the main reasons behind their projected changes [69]. In total, this additional information is available for approximately one-third of all occupations, but together they account for 70% of all projected changes in employment. This information is provided in the form of a textual explanation for each given occupation, and it contains details on the direction of change (negative or positive) and a description of the reasons behind this change. We categorize the range of reasons appearing in the BLS explanations as “related to technology” and “not related to technology.” Table 2 lists the types of reasons reported by the BLS, grouped by their relationship to technology. Column 2 of Table 2 provides some descriptions of the reasons driving employment projections. Column 3 shows examples of detailed reasons that fall into these categories. For some occupations, technologies create new tasks and responsibilities, such as handling increasing threats of cyber-attacks, analyzing digital data, or inventing new software and hardware. In other occupations, technologies change the nature of work. For example, some jobs are replaced by automation, AI, or robotics; some jobs based on traditional products or materials will be in lower demand as new products emerge (locksmiths are declining due to the adoption of smart locks); some jobs are in higher demand because of the deployment of new infrastructure (e.g., small commercial satellites and drones); some jobs are displaced because of internet-based platforms (e.g., ride-hailing apps, delivery apps).

**Table 2:** Reasons behind bureau of labor statistics employment projections for change in jobs by occupations.

Relationship to technology	Reason Category (coded from the detailed reasons)	Description of Reason from Bureau of Labor Statistics Projections
Related to technology	Need to manage risks, increase cyber security	Information Security Analyst (15–1212): Share increases as firms increase their IT security staff and capabilities as a response to increasing threats of cyber-attacks aimed at sensitive or financial data.
	Need to handle and/or analyze more/new data	Operations Research Analyst (15–201): share increases as larger amounts of digital and electronic data are collected over the next decade
	Need to develop new software/apps and/or hardware	Computer and Information Research Scientist (15–1221): share increases as demand for digital devices increases
	Need to develop and maintain tools to adjust to new technologies	Industrial Machinery Mechanics (49–9041): share increases as manufacturing automation increases requiring more maintenance and repair
	Job changes because of new infrastructures	Aerospace engineers (17–2011): share increases as aerospace engineers are needed in developing new technologies stemming from the wider commercial use of small satellites and drones.
	Job changes because of automation, AI, or robotics	Insurance claims and policy processing clerks (43–9041): share decreases as firms increase the use of computer software, enhanced with AI technology, to automate claims and policy processing.
	Job changes because of internet-based apps or platforms	Driver/sales workers (53–3031): share decreases as restaurant food delivery will increasingly be handled by delivery companies that utilize smartphone apps and classify workers as independent contractors.
	Job changes because of innovation or the emergence of products	Locksmiths and safe repairers (49–9094): share decreases as new smart locks, apps on smartphones, key kiosks, and voice–activated locks reduce the need to hire a locksmith to open locks or create key copies.
NOT related to technology	Organizational, industrial, economic restructuring	Cooks, Institution and Cafeteria (35–2012): share decreases as the use of food contractors continues to grow
	Specialization/higher demand for specialists (not due to tech)	Tree Trimmers and Pruners (37–3013): share increases as local governments desire more trees in urban areas
	Bad economic trends and decline in demand for specialists (not due to tech)	Carpet installers (47–2041): share decreases as other floor coverings replace carpet
	Offshoring, outsourcing, occupational substitution	Pharmacy Aides (31–9095): share decreases as pharmacy technicians, cashiers, and clerks take over duties traditionally done by aide
	Demographic change (including aging) and healthier lifestyle	Ophthalmic Laboratory Technicians (51–9083): share increases as an aging population means more demand for contact lenses and glasses

**Sources**: This table is based on BLS Employment Projections 2019–2029, Table 1.12 “Factors affecting occupational utilization, projected 2019–29”. Reasons are coded based on their own considerations.

For each of our clusters, we aggregate employment numbers across occupations with a BLS explanation, focusing on those related to technological change. These aggregated values allow us to quantify the share of jobs in each occupational cluster where technological change results in large gains or losses.

## Results

Table 3 provides information about the nested nature of the 17 occupational groups within the 8 macro clusters. Appendix D in S2 Appendix contains a detailed list of the occupations included in each of the cluster hierarchies.

**Table 3:** Clustering results based on ward’s method.

List of 8 clusters	List of 17 groups	Example occupations
Cluster 1: Managerial and supervisory occupations (n = 71)	Group 1: Managers (n = 43)	Human Resources Managers
		Sales Managers
		Agents/Business Managers of Artists/Athletes
		Transportation Planners
	Group 2: Supervisors (n = 28)	Postmasters and Mail Superintendents
		First-Line Supervisors of Retail Sales Workers
Cluster 2: Education, mental health and counselling occupations (n = 71)	Group 3: Educators (n = 39)	Biological Science Teachers, Postsecondary
		Communications Teachers, Postsecondary
		History Teachers, Postsecondary
	Group 4: Counselors (n = 32)	Clinical, Counseling, and School Psychologists
		Mental Health, Substance Abuse Social Workers
		Special Education Teachers, Secondary School
Cluster 3: Science, technology and engineering occupations (n = 100)	Group 5: Scientists (n = 36)	Biomedical Engineers
		Hydrologists
		Geographers
	Group 6: Non-medical technicians (n = 64)	Private Detectives and Investigators
		Sound Engineering Technicians
		Electrical/Electronic Engineering Technologists
		Agricultural and Food Science Technicians
		Computer Programmers
		Electrical and Electronics Drafters
Cluster 4: Special and support services occupations (n = 76)	Group 7: Medical technicians (n = 19)	Respiratory Therapists
	Group 8: Physicians (n = 36)	Adapted Physical Education Specialists
		Pharmacists
		Registered Nurses
		Oral and Maxillofacial Surgeons
	Group 9: Safety protectors (n = 21)	Police and Sheriff’s Patrol Officers
		Air Traffic Controllers
Cluster 5: Analytical and expert services occupations (n = 90)	Group 10: Analysts (n = 37)	Financial Examiners
		Statisticians
		Loan Officers
		Insurance Sales Agents
	Group 11: Clerical workers (n = 22)	Librarians
		Editor
	Group 12: Office staff (n = 31)	Medical Secretaries Data Entry Keyers
Cluster 6: Customer and personal services occupations (n = 86)	Group 13: Personal service providers (n = 55)	Home Health Aids
		Bakers
		Hotel, Motel, and Resort Desk Clerks
		Counter and Rental Clerks
		Cook, Fast Food
	Group 14: Sales persons (n = 31)	Costume Attendants
		Fitness Trainer and Aerobics Instructors
		Retail Sales persons
		Hairdressers, Hairstylists, and Cosmetologists
Cluster 7: Equipment operation and services occupations (n = 84)	Group 15: Equipment operators (n = 84)	Bus and Truck Mechanics, Diesel Engine Specialists
		Elevator Installers and Repairers
		Radio, Cellular, Tower Equipment Installers/Repairers
		Highway Maintenance Workers
		Crane and Tower Operators
Cluster 8: Basic services, crafts and manual occupations (n = 178)	Group 16: Craftspersons (n = 133)	Watch Repairers
		Fiberglass Laminators and Fabricators
		Industrial Truck and Tractor Operators
		Floor Sanders and Finishers
		Tile and Marble Setters
		Metal-Refining Furnace Operators and Tenders
	Group 17: Manual labors (n = 45)	Locker Room, Coatroom, Dressing Room Attendants
		Laborers and Freight, Stock, Material Movers, Hand
		Light Truck or Delivery Services Drivers

Our classification scheme illustrates the degree of human capital fluidity between similar occupations in the contemporary US labor market that is not confined to the boundaries of standard industry classification systems. For example, our Cluster 1 (Managers and Supervisors) includes many occupations that are listed under the “Management Occupations” major group of the SOC 2010 classification (code 11–0000), but it also incorporates occupations from other major groups of the SOC 2010 that are related to management in some specific industry or sphere. For instance, Cluster 1 includes “Directors, Religious Activities and Education,” which is listed under “Community and Social Service Occupations” major group in the SOC 2010 (code 21–0000), “Chefs and Head Cooks” which is listed under “Food Preparations and Serving Related Occupations” major group in SOC 2010 (code 35–0000), and “Supervisors of Production Workers” which is listed under “Production Occupations” major group in the SOC 2010 (code 51–0000). All these occupations have relatively similar requirements in terms of characteristics that we use in our analysis and are thus placed in the same cluster in our classification scheme. However, in the SOC 2010 classification scheme, they belong to different major categories or industries. Despite the optimized results derived from PCA clustering analysis, it should be noted that the clustering configuration and labeling may have errors or biases. Prior work has found that it lacks sound validation when performing unsupervised machine learning techniques to identify groups and using a supervised machine learning classifier (e.g., decision tree) to predict the cluster labeling [78].

### Cluster characteristics

Tables 4 and 5 contain summaries of the underlying occupational descriptors for 17 occupational groups, with the cluster structure delineated with shading. For educational attainment, experience, and training, O*NET reports the proportion of workers in an occupation that falls within each category: the level ranging from 1 (below high school) to 12 (post-doc) for education and the duration ranging from “0–3 months” to “10+ years” for experience and training. After clustering, we average the proportions of workers within each category of these variables for occupations falling in the same group and use these values as weights to compute the average education level and duration of experience and training for each occupational grouping. For values and interests, O*NET provides an expert assessment of the importance of 6 work values and 6 categories of work interests (ranging from 0–5, with 5 being very important) for a given occupation. These scores are averaged for occupations within each group and for visual convenience are rescaled to be between 0 (corresponding to 0 score) and 10 (corresponding to the highest average group score).

**Table 4:** The average education, experience and job training.

Cluster	Group	Education level (1–12)	Experience (years)	Job training (years)
C1: Managerial and supervisory occupations	G1: Managers	6.6	4.3	0.9
	G2: Supervisors	4.1	3.1	0.9
C2: Education, mental health and counselling occupations	G3: Educators	9.9	3.6	1.0
	G4: Counselors	7.8	2.2	0.8
C3: Science, technology and engineering occupations	G5: Scientists	7.3	3.4	1.2
	G6: Non-medical technicians	4.8	3.1	1.0
C4: Special and support services occupations	G7: Medical technicians	4.8	1.3	0.6
	G8: Physicians	8.4	2.5	1.0
	G9: Safety protectors	4.2	3.0	1.4
C5: Analytical and expert services occupations	G10: Analysts	5.7	2.7	0.9
	G11: Clerical workers	5.8	2.0	0.6
	G12: Office staff	3.7	1.7	0.6
C6: Customer and personal services occupations	G13: Personal service providers	2.6	1.0	0.5
	G14: Sales persons	2.7	1.3	0.6
C7: Equipment operation and services occupations	G15: Equipment operators	2.6	1.9	1.2
C8: Basic services, crafts and manual occupations	G16: Craftspersons	2.2	1.6	1.1
	G17: Manual labors	2.1	0.8	0.5

**Sources**: Authors own calculations. Occupations are clustered using the Ward hierarchical method based on 72 principal components derived from 220 occupational descriptors from O*NET. For each occupation, descriptors related to education, experience, and job training are aggregated into 3 corresponding variables and then averaged across occupations in each group.

**Table 5:** The average scores for work values and interests.

Cluster	Group	Work Values						Work Interests					
		Achievement	Independence	Recognition	Relationships	Support	Working Conditions	Artistic	Conventional	Enterprising	Investigative	Realistic	Social
C1: Managerial and supervisory occupations	G1: Managers	9	9	8	8	7	9	5	6	9	5	4	6
	G2: Supervisors	8	9	7	9	7	7	3	7	10	3	5	6
C2: Education, mental health and counselling occupations	G3: Educators	9	9	8	8	5	9	6	5	4	8	4	10
	G4: Counselors	9	8	8	10	7	8	6	5	5	6	3	10
C3: Science, technology and engineering occupations	G5: Scientists	9	8	8	6	7	8	4	6	4	10	8	3
	G6: Non-medical technicians	7	8	7	6	8	7	4	7	4	7	8	3
C4: Special and support services occupations	G7: Medical technicians	7	7	6	9	9	7	2	6	4	6	8	8
	G8: Physicians	9	9	8	10	7	8	3	4	4	8	7	9
	G9: Safety protectors	8	8	7	8	9	7	2	6	7	5	9	5
C5: Analytical and expert services occupations	G10: Analysts	8	8	7	7	7	7	3	9	8	5	3	4
	G11: Clerical workers	8	7	6	8	6	7	7	5	6	5	3	6
	G12: Office staff	5	6	5	8	7	5	2	10	7	3	4	4
C6: Customer and personal services occupations	G13: Personal service providers	5	6	4	8	7	5	2	8	6	3	8	5
	G14: Sales persons	6	6	5	8	5	5	5	6	7	2	7	5
C7: Equipment operation and services occupations	G15: Equipment operators	5	6	4	6	8	6	2	6	3	4	10	2
C8: Basic services, crafts and manual occupations	G16: Craftspersons	4	5	4	6	7	5	2	6	3	4	10	2
	G17: Manual labors	3	4	3	6	6	4	2	7	4	2	9	3

**Sources**: Authors own calculations. Occupations are clustered using Ward hierarchical method based on 72 principal components derived from 220 occupational descriptors from O*NET. Descriptors related to Work Values and Work Interests are averaged across occupations in each group and re-scaled to be in the [1–10] interval.

Cluster 1 comprises groups one and two containing management and supervisory occupations. Group 1 includes managers in producer services (e.g., business and financial operations), and Group 2 contains preschool education administrators, chefs and head cooks, residential advisors, and first-line supervisors. These occupations require knowledge in administration and management as well as personnel and human resources. They also require social skills, including negotiation, coordination, persuasion, and complex problem-solving skills. Cluster 2 is comprised primarily of post-secondary education occupations (Group 3) as well as occupations in mental health and counseling (Group 4). These occupations require knowledge in education and training as well as psychology, therapy and counseling. Occupations in Cluster 2 have the highest level of educational attainment: 29% of occupations have a Master’s degree, 34% have a Ph.D., and 9% have some sort of post-doctoral experience.

Cluster 3 contains engineers, computer programmers, technicians, and other occupations where data and analysis are at the core of their daily activities. These occupations require knowledge of the sciences (chemistry, biology and physics), computers and electronics, and engineering and technology. The occupations also require various technical skills, including complex problem-solving, programming, design, troubleshooting, and operations analysis. Cluster 4 contains occupations that require knowledge across a range of domains, including biology, medicine and dentistry, public safety and security and law and government. These occupations require skills in science, complex problem solving and critical thinking, as well as abilities related to problem recognition or problem sensitivity. This cluster has a great deal of variation in educational requirements: 22% of occupations have an associate’s degree, 18% have a bachelor’s degree, 10% have a Master’s degree, 13% have a doctorate, and 8% have post-doctoral education. Occupations in Cluster 5 primarily include analytical, clerical, and office jobs, which deal with data, writing, and paperwork as their core activities. These occupations are similar to Cluster 3 in their skills (e.g., mathematics, critical thinking, complex problem solving) and abilities (e.g., mathematical reasoning, number facility). However, occupations in Cluster 5 involve more structured work than in Cluster 3.

Cluster 6 represents customer and personal services occupations, such as food preparation, retail salespersons, and hairdressers. Cluster 7 groups equipment operation and service occupations, such as bus mechanics, elevator installers, and crane operators. Cluster 8 includes basic services, crafts, and manual occupations, such as watch repairers, material movers, and tile setters. Tables 4 and 5 illustrate that Clusters 6, 7 and 8 are distinct from the other five clusters discussed to this point in time with respect to their levels of educational attainment and work type. Workers in these occupations only require some amount of post-secondary education. In terms of work values, these clusters have a lower average score in achievement, independence, recognition, and work conditions, while having higher average scores on support (as well as relationships in some service-related groups). Work interests in these clusters tend to be relatively more conventional and realistic compared to Clusters 1–5. Cluster 6 is comprised primarily of service occupations that require knowledge about food production and customer and personal service. Clusters 7 and 8 are similar in that they share realistic interests with practical, hands-on work. These occupations often involve working outside with tools and machinery or plants and animals. The majority of occupations in Cluster 7 require mechanical knowledge and technical skills related to equipment maintenance, equipment selection, and repair. Cluster 8 contains occupations that require substantial manual labor and knowledge of the following areas: transportation, production and processing, and customer and personal service. They also require a high degree of technical skills related to equipment selection and maintenance, as well as social skills.

### Wage patterns in clusters

**Fig 2:**
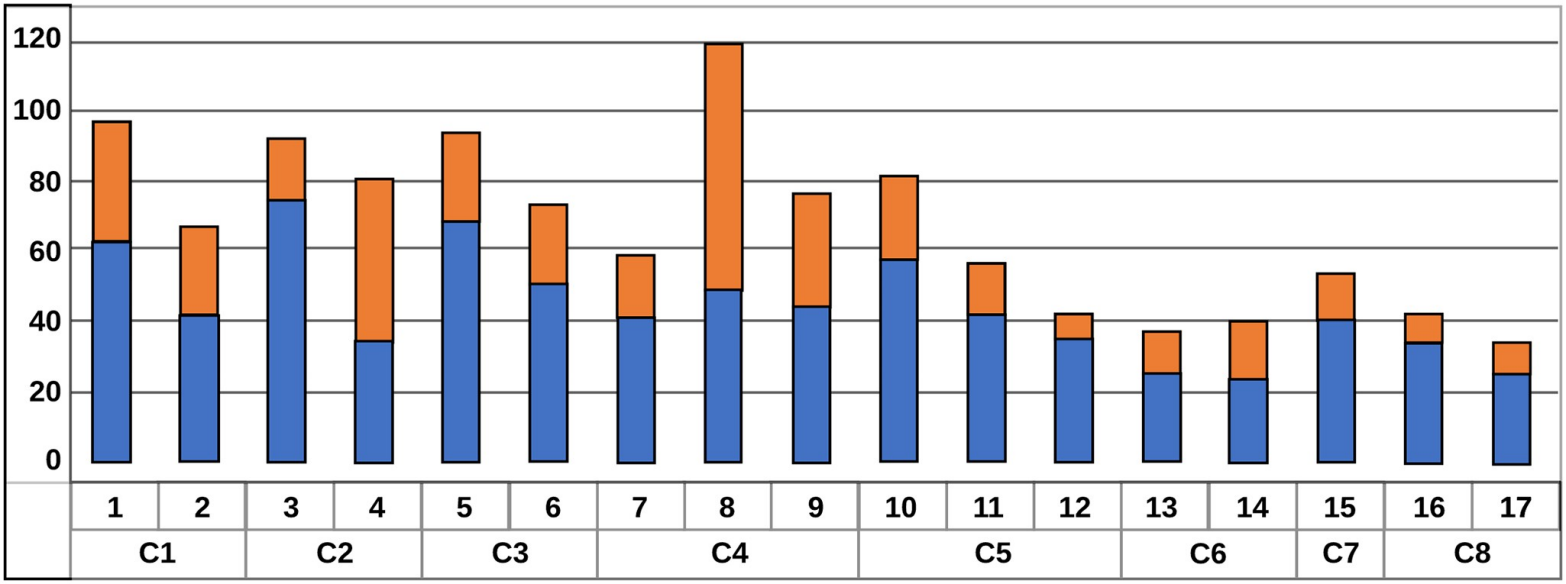
The mean and the standard deviation in wages by occupational groupings. **Sources**: Based on wage data from the Occupation Employment Statistics survey (BLS), occupations are clustered using the Ward hierarchical method based on 72 principal components derived from 220 occupational descriptors from O*NET. The dots represent the mean wage for each sub-group, and the bars reflect the standard deviations in wages for the given sub-group.

Fig 2 displays information about the mean and standard deviation of the mean annual wage for each of the seventeen groups developed for this study. Appendix E in S2 Appendix contains more detailed information about the mean annual wage, standard deviation, and coefficient of variation for these groups. Groups 1, 3, 5, and 8 have the highest incomes, with a mean wage of at least $80,000. These groups are shaded in green. Examples of highly paid jobs in Group 1 include management occupations with a wage somewhere around $105,000. Group 3 contains primarily education administrators. Group 5 is composed mainly of engineering occupations which are known to be high-paying jobs. The highest mean wage, of about $120,000, is observed in Group 8, which contains some highly paid medical occupations (i.e., orthodontists, surgeons, anesthesiologists). Incidentally, this is also the group with the highest variation relative to the mean (the coefficient of variation in this group reaches 58%). The high coefficient of variation reflects lower wages of some counseling jobs in this group (marriage and family therapists, for example, with an average wage of $55,000 per year) and much higher-paid counseling jobs that require a medical background (for example, psychiatrists earn $178,000 per year).

There are several occupational groups shaded in yellow (Groups 1, 4, 6, 7, 9, 10, 11, 15) that make average wage levels falling somewhere between $50,000 and $80,000. Group two contains a lot of managerial and supervisory positions, while Group 4 contains a lot of teaching, counselors and social workers. Group 9 contains a lot of first responder workers (e.g., paramedics, police officers, firefighters) and occupations working in aviation and nuclear power. Group 15 contains technicians, repairers, and other types of specialized workers (e.g., hazardous materials removal workers).

Groups 12, 13, 14, 16 and 17 contain occupations with below-average wages. Occupations in these groups make between $30,000 and $45,000. Group 12 contains several clerical and secretarial positions, including paralegals and legal assistants. Group 13 contains various low-paid service occupations, including fast food servers, maids and cleaners, cashiers, shipping clerks, and other similar professions. Group 17 contains manual labor occupations (e.g., quarry rock splitters, sewing machine operators, cutters and trimmers and other similar occupations).

### Employment trends in clusters

**Fig 3:**
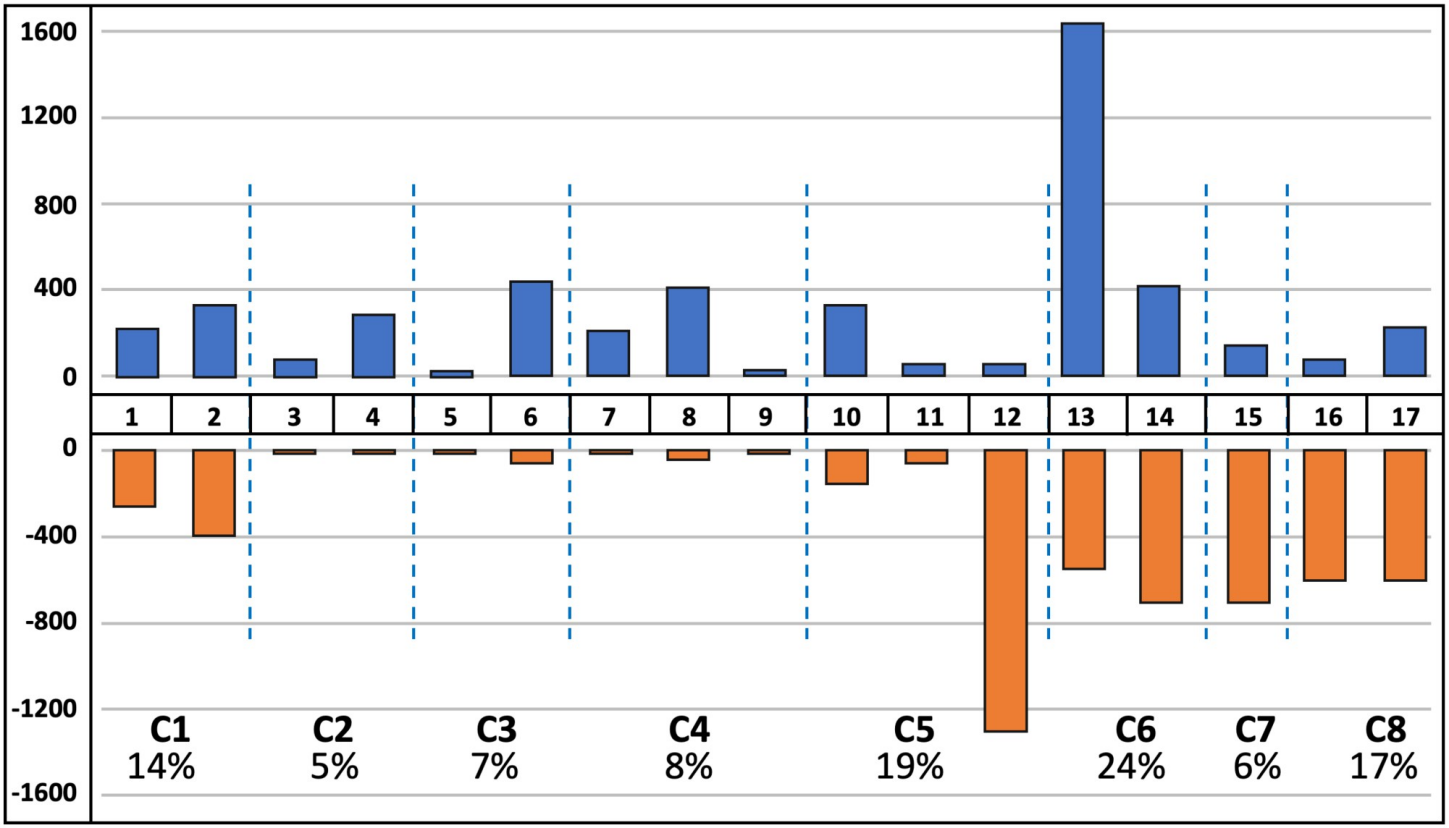
Job losses and gains by occupational groupings (in thousands). **Sources**: Based on BLS Employment Projections 2019–2029, Table 1.2 “Employment by detailed occupation, 2019 and projected 2029”. Occupations are clustered using Ward hierarchical method based on 72 principal components derived from 220 occupational descriptors from O*NET. Gains are shown in blue, and losses–in orange.

To evaluate the employment trends within occupational groups, we turn to EP data from the Bureau of Labor Statistics. Fig 3 displays the results for the 17-group solution, with detailed statistics provided in Appendix F in S2 Appendix.

There are large differences in cluster sizes based on the number of jobs within them, with Cluster 6 alone, which is the largest cluster, accounting for nearly one-quarter of all jobs in 2019 –as much as the smallest 4 clusters (2, 3, 4 and 7, which account for 5–7% of total employment each). The medium-sized Clusters 1, 5, and 8 contain 15–20% of all jobs each. As discussed above, Clusters 6–8 differ in their human capital requirements and work interest and values, and now we see that together these clusters account for nearly half of all jobs. Group 12 shows many patterns similar to those observed in Clusters 6–8 (by wages and human capital requirements). If we add this group to Groups 13–17 (Clusters 6–8), together, they account for almost 60% of all jobs.

Fig 3 shows that the largest gain in jobs is projected for Cluster 6, especially in Group 13, which has the largest net gain of occupations, despite also having sizable job losses. Most gains in this group are due to expansion in personal assistance-related jobs (e.g., home health aides, physical therapist aids) and jobs related to fine dining (e.g., restaurant cooks, bartenders). Otherwise, job gains are relatively evenly spread across the remaining clusters.

As for job losses, these are mostly concentrated in Groups 1 and 2 (Cluster 1) and in Groups 12–17. Losses in Cluster 1 are more or less compensated with gains of similar size within each group. This indicates that although there will be significant churn or internal mobility within these groups, most changes will not force workers out of the groups, implying relatively low costs of mobility. Instead, in several groups with large job losses, there are no gains of comparable size to accommodate internal mobility, so there will be a push for workers to move out of their occupational “neighborhood” and face a higher cost of mobility. This is especially pertinent for groups 12 and 16, which have large net losses in jobs. Moreover, the job base is narrowing not only within this group but also in many similar groups, which could further constrain occupational mobility.

**Fig 4:**
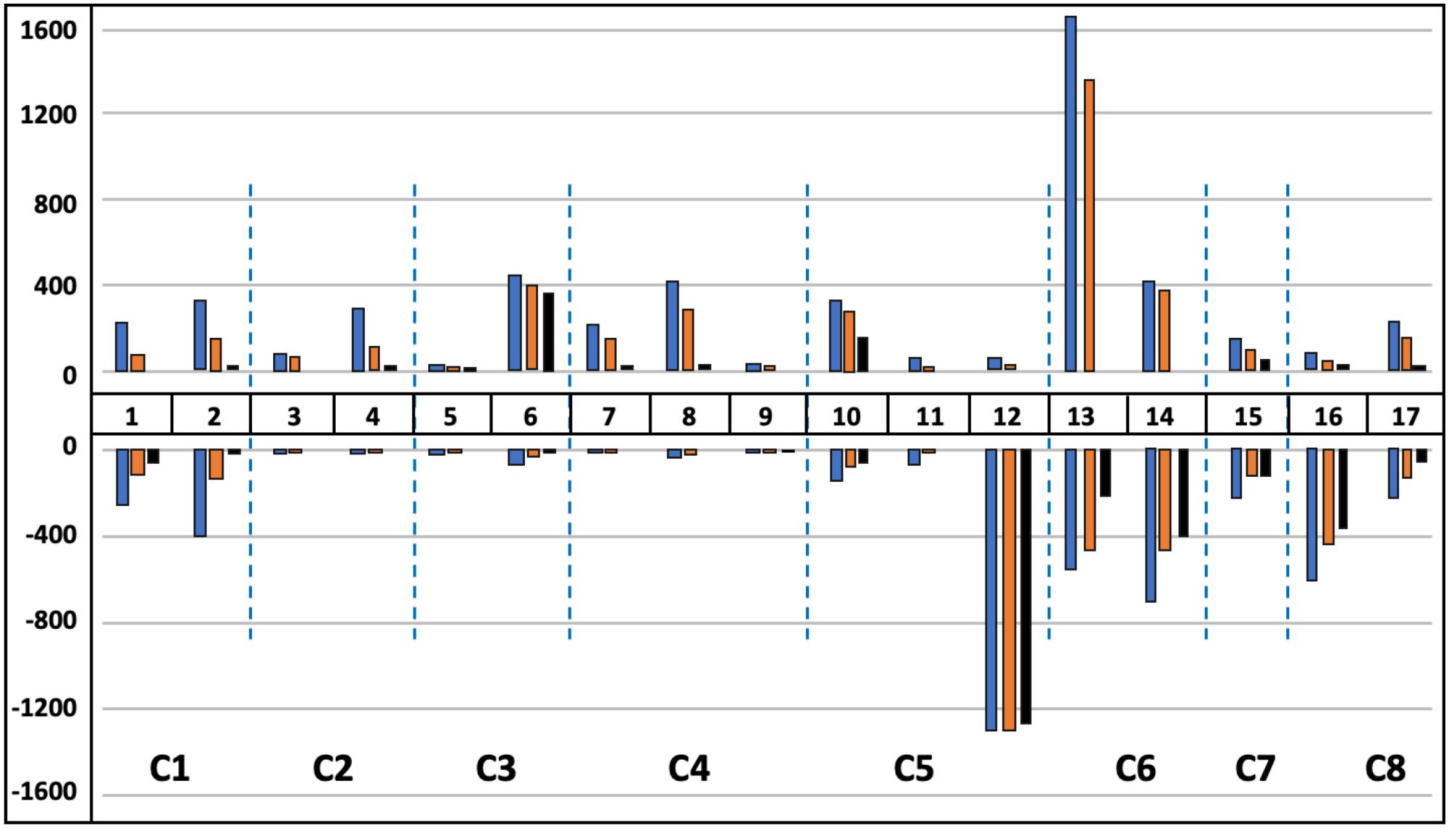
Job losses and gains by occupational groupings, including technological factors. **Sources**: Based on BLS Employment Projections 2019–2029, Table 1.2 “Employment by detailed occupation, 2019 and projected 2029” and Table 1.12 “Factors affecting occupational utilization, projected 2019–29”. Occupations are clustered using the Ward hierarchical method based on 72 principal components derived from 220 occupational descriptors from O*NET. Total change is shown in blue, change that has a reason is shown in orange, and change due to technology is shown in black.

Fig 4 demonstrates the role of technology in job dynamics by occupational groups. Appendix G in S2 Appendix provides the supporting statistics. The figure highlights that job losses due to technological change are mostly concentrated in Groups 12–17. Technology accounts for half of all jobs lost in Clusters 6, 7, and 8 and for nearly 90% of jobs lost in Cluster 5 (mostly concentrated in Group 12). Alternatively, Clusters 1–4 appear to be robust to job losses due to technological change. In particular, Clusters 2 and 4, which contain postsecondary education occupations and occupations in science, law, and government, are projected to grow somewhat. This growth is due to other non-technology-related factors (e.g., increased specialization). In Cluster 3, which is comprised of engineers, computer programmers, and technicians, three-quarters of job growth is due to technology.

**Fig 5:**
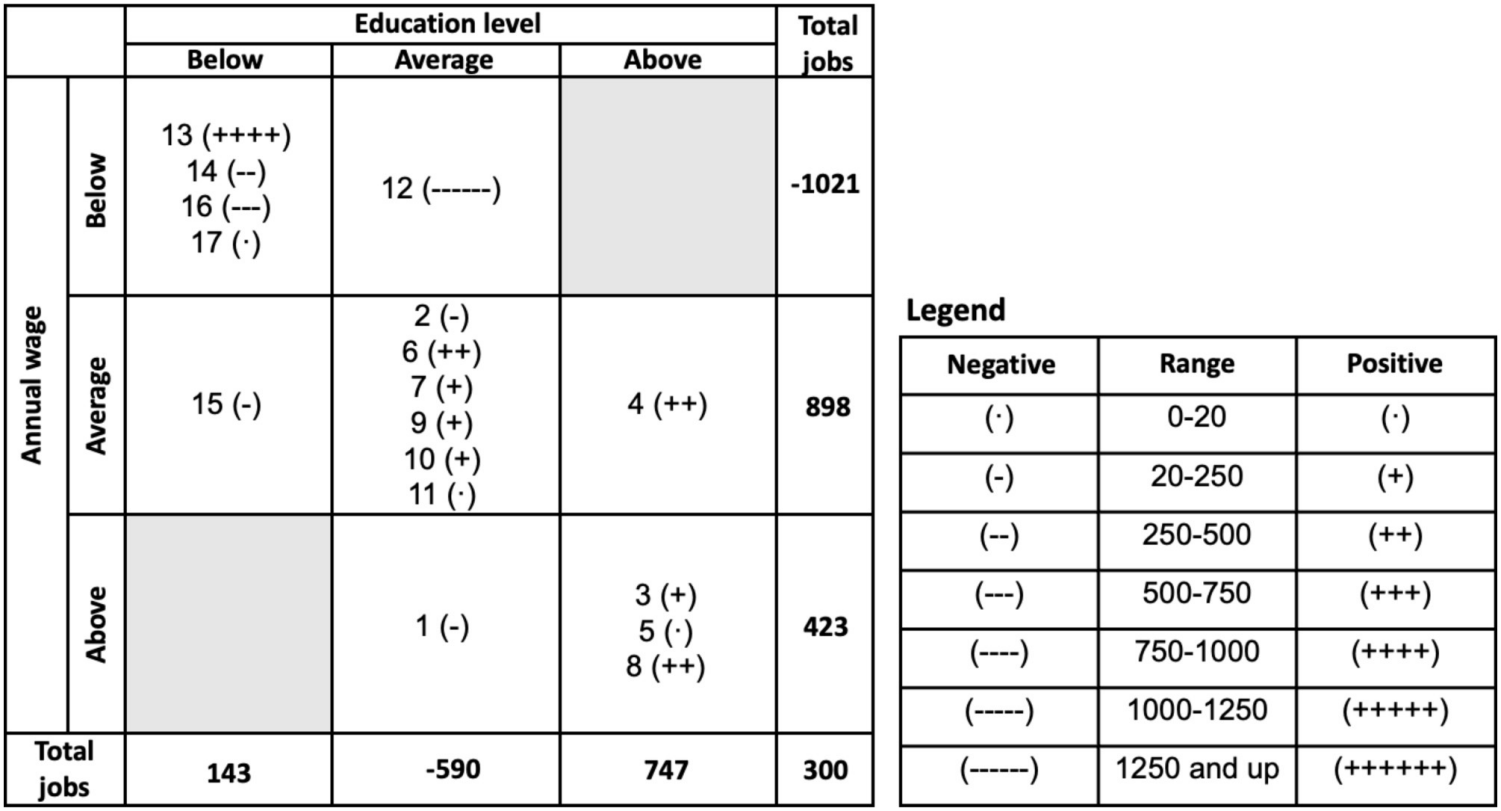
Job losses and gains by occupational groupings—By wage and education level. **Sources**: Authors calculations based on BLS Employment Projections 2019–2029, Table 1.2 “Employment by detailed occupation, 2019 and projected 2029”. Occupations are clustered using the Ward hierarchical method based on 72 principal components derived from 220 occupational descriptors from O*NET.

Fig 5 summarizes three aspects of clusters in terms of their employment trends, educational attainment, and wages. It arranges occupational groupings in a 3x3 matrix where the columns show three categories of education: 1.5 standard deviations below the overall mean of education level (“Below” column including education levels ranging from below high school to post-secondary), 1.5 standard deviations within the overall mean education level (“Average” column, which includes some college, associate degree, BA degree or post-BA certification), and 1.5 standard deviations above the overall mean (“Above” column with education levels ranging from MA diploma to post-doc). The rows are designed in a similar way showing departures from the overall mean annual wage. The net gains and losses in jobs within each group are reflected with minus/plus symbols, with the number of symbols reflecting the absolute size of the net change. In addition, the column sum at the bottom of this figure indicates the net gain in jobs by education level, and the row sums indicate the net gain in jobs by wage level.

A general picture that emerges from examining the row and column totals in Fig 5 suggests the following two processes taking place in the job market. First, there is an expansion in occupations that require below or above-average levels of education, while the demand for jobs described by the average level of education will decrease. Second, occupations that are currently paying low wages are projected to shed many jobs, while occupations with average to high wages are going to expand. This massive migration of jobs into higher paying but relatively distant in terms of human requirements and work type occupational groups could be a costly process. They could require time and assistance from policymakers and human resource specialists. In the short run, it could also mean excess labor in occupational groups with projected job losses, possibly depressing wages paid in these occupations, and an increased probability and duration of unemployment. Instead, in the expanding occupational groups, there could be short-run labor shortages, fueling up already high wages.

A more detailed examination reveals a large portion of gains will come from jobs that have above-average wages and levels of educational attainment (e.g., Groups 3 and 8). Group 3 contains various types of post-secondary educators (e.g., physics, chemistry, political science). Growth in this group likely stems from anticipated increases in undergraduate enrollment at institutions of higher education; by 2029, undergraduate enrollment is projected to increase by 17 million students (NCES, 2020). The educational attainment for both of these occupational groups is substantial. A total of 93% of occupations in Group 3 and 62% of occupations in Group 8 require a Master’s degree or higher. Group 8 contains many medical support occupations, such as health educators, pharmacists, and optometrists, as well as surgeons and anesthesiologists. The growth in this cluster is not surprising, given the anticipated need for medical professionals. As the population ages and people live longer, many will need treatment for chronic health conditions [79].

Job growth is anticipated in occupations that require average levels of educational attainment and pay average wages. Most of the growth in this category is in Group 6, which contains science, technology and media specialists and technicians. A total of 73% of occupations in this group require education beyond high school and up to (but not exceeding) a bachelor’s degree. In this group, 80% of the jobs created will be due to technological change. The EP data note the reasons behind this technology connection. For example, more jobs will be needed in cybersecurity to protect sensitive financial and personal data as an increasing amount of information is stored in cloud environments. More jobs will be needed to design, create and implement software to run all the devices connected to the Internet as society moves towards an Internet of Things (IoT). Job growth in Group 10 is also related to the digitization of society, as more people working in occupations that analyze data will be needed to enhance business processes and new products.

Groups with job losses (e.g., 12, 14, 15, 16) are clustered in the lower left cells of Fig 5 and have lower levels of educational attainment and wages. As discussed, these groups will lose a large portion of jobs because of technological change. In fact, for some, such as Group 12, almost all job losses are linked to technological change. Examples of jobs that will be lost include paralegals and legal assistants, new accounts clerks, correspondence clerks, and medical secretaries. These jobs are expected to be replaced by capital as automation enables machines to do the jobs of people or enables fewer workers to perform their jobs more efficiently. Group 16 is projected to lose a large number of jobs due to technological change as well. These job losses are related to capital/labor substitutions. For example, hand packers and packagers are projected to be replaced by machines that will do the same work. Another large chunk of jobs will be lost due to productivity changes. In the future, for example, fewer workers in the job of “Tool Grinders, Filers, and Sharpener” will be needed as programmable equipment will help them to perform their jobs more efficiently.

Three groups that run counter to the trends described above are Groups 1, 2, and 13. In Group 1, which pays above-average wages but requires average levels of educational attainment, technology is expected to replace the following types of jobs: purchasing managers, sales managers, buyers and purchasing agents, and logisticians. This job replacement is projected to be a result of task automation as capital substitutes for labor. These job losses are of concern because of their high wage levels; workers in these occupations may be forced to transition to other occupations that pay less. Group 13 is projected to experience an increase in jobs. Unfortunately, these gains are not necessarily advantageous to workers because of their below-average educational requirements and low wages. Examples of occupations in this group include fast food cooks, restaurant servers, occupational therapy aides, and pharmacy aids. Thus, workers in the occupations may need to work multiple jobs to maintain a particular standard of living.

## Discussion

Innovations in technology, including the Internet of Things (IoT), artificial intelligence, autonomous vehicles, and drones, are reshaping the workplace. These innovations are changing the types of work being done and how work is done [80–83]. Technological change has also visibly impacted the labor market as jobs become increasingly bifurcated into low-skill, low-wage jobs and high-skill, high-wage jobs [2, 84, 85]. This bifurcation of work has important implications for the ability of workers to change jobs or occupational mobility.

To this point in time, empirical work related to routine-biased technological change (RBTC) has produced important information about the costs of mobility for older workers [86] and declining wage premiums for routine jobs in manual labor [11]. To our knowledge, studies do not consider the impact of technological change on the size of job clusters prior to analyzing occupational mobility. This a priori step is important because technological change may eliminate several jobs within occupational clusters, limiting the number of occupational alternatives for workers. Our study addresses this knowledge gap by undertaking a comprehensive clustering of over 700 occupations across multiple dimensions (e.g., KSA, human capital, worker values and interests) to create groups of similar jobs. We presented information on wage variations within occupational clusters to demonstrate their coherence. After summarizing the characteristics of occupational clusters, we analyzed their growth trajectory and assessed the role of technological change on job dynamics. This analysis is insightful for future work assessing occupational mobility since movement between jobs within the same cluster, particularly for finer-resolution cluster solutions, is likely easier than movements between clusters.

Consistent with prior work, analytical results revealed technological change is responsible for a large proportion of job losses in low-wage, low-skill occupations (e.g., Groups 12, 14, 16) [87]. Occupations with job losses had the characteristics of jobs anticipated to be disrupted by technology; routine and somewhat repetitive in nature [11, 12]. Group 12 was a good example of technology replacing workers or enabling businesses to continue operations with fewer workers. The analysis also indicated continued growth of high-wage, high-educational attainment jobs requiring a Master’s degree or higher (e.g., post-secondary educators and medical support occupations), as well as high-wage occupations where task automation enables capital to substitute for labor (e.g., purchasing managers, logisticians). Losses in these occupations are less of a concern however because they also require high levels of educational attainment which indicates these workers will be able to find alternative forms of work. The projected large loss of jobs in low-wage groups (e.g., Groups 12, 14, 16) is cause for concern. This will limit alternative jobs that are closest to the lost jobs in terms of educational levels, skills, interests, values etc. and means that workers in these highly impacted groups will likely need to be retrained and/or go back to school in order to move to jobs outside of their occupational group.

The findings also indicated growth of jobs in occupations paying average wages and requiring average levels of educational attainment (an education beyond high school and up to, but not exceeding a college degree). Much of this job growth is related to Internet-related technologies and the Internet of Things (IoT). Moving forward, it will be important to track the wage levels of these high-growth occupational groups to ascertain if wages in these groups decline as more workers fill these positions. If wages do decline, this will certainly indicate a continued trend of disappearing jobs in the middle of the wage spectrum and continued wage inequality in the workforce related to technological change and RBTC [88, 89]. At this point in time, however, our results do not suggest, as RBTC does, that jobs in the middle portion of the wage spectrum will disappear by 2029. This a short-run outcome, however. Wages in these occupations will need to be tracked long-term to ascertain if they grow, decline or stay the same.

Our analytical approach makes several contributions to work on technological change and occupational mobility. First, it provides a comprehensive, multidimensional perspective on occupational grouping, which moves beyond the prior unidimensional emphasis of prior work on either skills, knowledge, tasks, or worker values and interests. Second, our clustering approach created a multi-level taxonomy of similar occupations that provide both macro-level snapshots and detailed micro-level information that may be used in workforce development and training initiatives. Three, our work provides a detailed taxonomy of similar occupations at finer levels of classification (provided in Appendix D in S2 Appendix), which can be of great practical use. For example, human resources specialists and counselors can use this information to advise workers on decisions related to occupational choice and mobility. Four, our analysis assesses the impact of technological change on job dynamics within clusters, which is important for assessing future employment alternatives for workers displaced by technological change.

That said, it is essential to acknowledge some limitations of our work, which relate to assumptions underlying the analysis. Specifically, we assume that the structure of clusters is static, which implies that the characteristics used to cluster occupations will remain unchanged over time. While this assumption is naive if one takes a long-term perspective, for short- to medium-term analyses, this assumption is not unreasonable. Another assumption is that job gains or losses within occupations where technology was indicated as the primary source of change were fully attributed to technology. In reality, technology might not be the only reason for observed changes. This situation was not very common, however, so we do not expect it to affect our main findings. Our analysis is based on pre-COVID-19 projections, so it is not fully reflective of changes in the labor market that might have been triggered by the effects of the pandemic. Additionally, our analysis does not assess occupational mobility between clusters. Instead, the taxonomy developed in this study may be used to assess movement between occupations in future work. Lastly, people need to be cautioned that the implementation of clustering and interpretation of results may have errors or biases due to the limitation of the algorithm [78, 90]. Interpretation and Implications of the results should be paired with theories and the literature.

From a policy perspective, the taxonomy of occupations and their projected growth provides critical information for policy makers to focus attention on particular segments of the taxonomy projected to experience the greatest pressure from technological change. In this respect, our study provides a foundation for moving forward in a proactive way for worker retraining in several ways. First, our nested occupational clustering provides a foundation for developing worker training and certificate programs by identifying knowledge, skills, and educational gaps between declining and growing occupations. Second, the information about values and interests provides people with a sense of the occupations that are most aligned with their values and interests, which could increase worker satisfaction after moving to alternative occupations.

Another dimension for targeted policymaking involves dedicated attention to groups most vulnerable to technological change. Older workers, for example, might be less likely to invest in acquiring new types of skills and knowledge [91] and might be less adaptable to changes in work due to physical constraints and established habits [92]. Older workers may also have more difficulty adapting to new technology compared to younger workers [93], which means that older workers employed in occupations most affected by technological changes could be facing a higher risk of labor market displacement and higher associated costs. Another example of a vulnerable population is ethnic and racial minorities, who are more likely to be concentrated in occupations associated with low skill requirements and low wages [94]. Racial/ethnic minorities are also likely to experience more labor market discrimination in times of economic scarcity [95], including the scarcity of jobs in relevant occupational groups.

For vulnerable groups, on-the-job training may be the best means of skills upgrading. For a long time, employers in the United States have not done a good job at providing on-the-job training [61, 62] and studies point to the need for a turning of the tide for displaced workers but also employers who are finding it increasingly difficult to find qualified workers for open positions [96]. On-the-job training may mean hiring workers without college degrees and providing training in skills where companies need workers. This practice would line up with emerging corporate practice, which sees employers moving towards hiring people without four-year college degrees for entry-level positions, particularly for racial/ethnic minorities [97]. Community college programs may also be a means of helping the workforce gain technology-relevant skills in shorter amounts of time, which may be critical as the pace of technological change quickens. Pima Community College in Arizona, for example, has a one-semester certificate program called “Autonomous Vehicle Driver & Operations Specialist,” which trains drivers on how to operate and interact with self-driving trucks [98].

Finally, the issue of inequality in educational opportunities has been an increasing concern and exacerbated by the rapid pace of technological advancement, as access to and proficiency with technology has become increasingly important in the workforce [63, 64]. Our analyses of wage patterns and projected employment trends within occupational clusters highlight the urgent need for policy interventions aimed at narrowing the technological gaps in educational opportunities. To effectively address this issue, policymakers must focus on improving access to technology and digital literacy skills for all students throughout childhood and young adulthood. This can be achieved through initiatives such as providing public schools (especially those in the underrepresented neighborhoods) with more resources for technology infrastructure and training teachers in digital education strategies. Additionally, efforts should be made to increase access to technology outside of the classroom, such as through community educational centers and public libraries.

## Conclusions

Technological change will continue to have major effects on the development of the labor market. As technology replaces jobs or changes the nature of work, people may have to transition into new positions. This paper provided new knowledge to the literature on technological change and occupational mobility by classifying occupations across a range of characteristics, including knowledge, skills and abilities (KSA), human capital requirements (education, experience, and training (EET)) and work activities, values and interests (AVI). The framework behind this classification is that movement between jobs within the same cluster is easier than movement between clusters. We then analyzed the anticipated impact of technological change on job growth in these clusters. This analysis revealed that large portions of clusters will be lost because of technology, which poses occupational mobility challenges for workers in these jobs at present. The analysis also revealed losses in low-wage, low-skill jobs and growth in mid-skilled and highly skilled jobs, which suggests a critical need for skills upgrading and workforce development initiatives in the next ten years to prevent job displacement of workers in low-wage occupations. Our findings complement the classic theory of routine-biased technological change, suggesting that many mid-wage, routine-based occupations will continue growing because of technologies in the short run. Special attention should be paid to vulnerable groups of workers concentrated in occupations with jobs most threatened by technological changes, including but not limited to older individuals and representatives of ethnic and racial minorities.

## Supporting information

S1 Appendix Technical appendix. (DOCX)

S2 Appendix Appendices. (DOCX)
