# Supplementary material for: Assessing the impact of technological change on similar occupations: Implications for employment alternatives
Source: PLoS One. 2023 Sep 18;18(9):e0291428. doi: 10.1371/journal.pone.0291428 (PMC10506722; doi:10.1371/journal.pone.0291428)
Supplement: S1 Appendix — (DOCX) [file pone.0291428.s001.docx]

**Assessing the Impact of Technological Change on Similar Occupations: Implications for Employment Alternatives**

## **S1 Technical Appendix**

Ward’s hierarchical clustering approach is used to cluster 756 occupations (2018 SOC) from O*NET based on information on the knowledge, abilities and skills requirements (KSA), the required education, experience, and training (EET), work activities, values and interests (AVI). Using the Duda-Hart index (Figure TA1), the following taxonomy of occupations is constructed:

- Level 1: 8 Occupational Clusters
- Level 2: 17 Occupational Groups
- Level 3: 40 Occupational Sub-groups
- Level 4: 59 Occupational Micro-groups

Duda-Hart statistic rises above the value of 0.9 in 3 cases: for 17, 40, and 59 cluster splits. In addition, for 8 cluster splits, the value of the DH statistic is a local maximum with a value of 0.89.

**S1 Figure TA1. Duda-Hart Index and Points of Local Maxima (Index Value > 0.9 and 8-Group Split)**


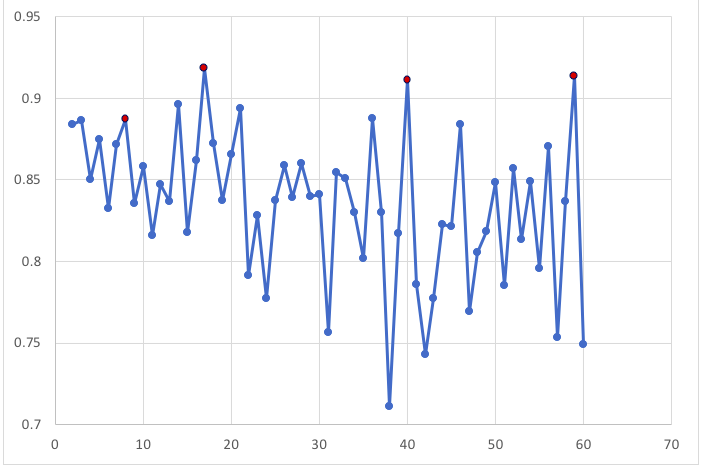


**Sources**: Based on clustering using Ward hierarchical method based on 72 principal components derived from 220 occupational descriptors from O*NET.

Table TA1 provides the goodness-of-fit measure based on wages for the four levels of classification. The fit improves when moving to a finer classification level, reaching 72-74% for 40 and 59-group solutions. This level of fit suggests that our cluster solutions lead to compact groupings of occupations, especially at finer levels of classification.

**S1 Table TA1. Sums of Squares within Groups and Goodness of Fit**

| **Level of clustering** | **SSW** | | | **Goodness of Fit** |
| --- | --- | --- | --- | --- |
|  | **Actual** | **Lower bound** | **Upper bound** |  |
| c=1 | 552,280 | 13,256 | 883,236 | 0.38 |
| c=2 | 448,671 | 12,946 | 875,489 | 0.49 |
| c=3 | 247,763 | 12,618 | 852,022 | 0.72 |
| c=4 | 226,072 | 12,397 | 836,777 | 0.74 |

**Sources**: Mean annual wage data (in thousand USD) are from the Occupation Employment Statistics survey (BLS); occupations are clustered using Ward hierarchical method based on 72 principal components derived from 220 occupational descriptors from O*NET. Own calculations.

Appendix D provides the full listing of occupations and their taxonomy for all four levels of classification. The average size of clusters and groups is 95 and 44 occupations, respectively. Sub-groups have 19 occupations on average, while the average micro-group size is 13 occupations.

The finest level of classification might be useful for various practical purposes, for example, for exploring and comparing careers by job seekers, workforce development and HR professionals. Here we illustrate an example of a micro-group and provide some insights behind occupational grouping at this level. Table TA2 lists the 14 occupations within micro-group 59.

**S1 Table TA2. List of 14 Occupations in Micro-Group 59**

| **SOC Code** | **Title of the occupation** |
| --- | --- |
| 39-3021 | Motion Picture Projectionists |
| 43-5021 | Couriers and Messengers |
| 43-5041 | Meter Readers, Utilities |
| 43-5052 | Postal Service Mail Carriers |
| 43-9071 | Office Machine Operators, Except Computer |
| 49-3022 | Automotive Glass Installers and Repairers |
| 49-9091 | Coin, Vending, and Amusement Machine Servicers and Repairers |
| 51-9151 | Photographic Process Workers and Processing Machine Operators |
| 53-3031 | Driver/Sales Workers |
| 53-3033 | Light Truck or Delivery Services Drivers |
| 53-3052 | Bus Drivers, Transit and Intercity |
| 53-3058 | Taxi Drivers and Chauffeurs |
| 53-6011 | Bridge and Lock Tenders |
| 53-6021 | Parking Lot Attendants |

**Source:** occupations are clustered using Ward hierarchical method based on 72 principal components derived from 220 occupational descriptors from O*NET.

Several occupations in this micro-group are transportation-related whether that involves driving as a core component of job activities (e.g., meter readers, utilities; bus drivers, transit and intercity) or work facilitating the transport of people or products. For example, bridge and lock tenders (SOC code 53-6011) operate machinery on canals, railroads, drawbridges and any other type of adjustable bridge to control the flow of traffic whether that is boats, cars, trucks, people or railcars. Several of these transportation-related jobs also have a customer and personal service aspect to them. This explains why jobs that are not necessarily transportation-related are included in this cluster: Coin, Vending, and Amusement Machine Servicers and Repairers; Motion Picture Projectionists; Automotive Glass Installers and Repairers; Photographic Process Workers and Processing Machine Operators.

**S1 Table TA3. The Average Education, Experience and Job Training**

| **Classification** | | | | **Av. Educ. Level (1-12)** | **Av. Exper. (years)** | **Av. job training (years)** |  | **Classification** | | | | **Av. Educ. Level (1-12)** | **Av. Exper. (years)** | **Av. job training (years)** |
| --- | --- | --- | --- | --- | --- | --- | --- | --- | --- | --- | --- | --- | --- | --- |
| **Cluster** | **Group** | **Sub-group** | **Micro-group** |  |  |  |  | **Cluster** | **Group** | **Sub-group** | **Micro-group** |  |  |  |
| **1** | **1** | **1** | **1** | 7.1 | 5.5 | 1.1 |  | **5** | **10** | **20** | **29** | 6.6 | 3.5 | 1.5 |
|  |  |  | **2** | 6.0 | 4.8 | 0.7 |  |  |  |  | **30** | 8.9 | 2.0 | 0.8 |
|  |  |  | **3** | 6.9 | 3.9 | 0.8 |  |  |  | **21** | **31** | 5.4 | 2.8 | 1.0 |
|  |  | **2** | **4** | 6.5 | 3.3 | 1.1 |  |  |  |  | **32** | 4.5 | 2.0 | 0.9 |
|  | **2** | **3** | **5** | 4.6 | 2.9 | 0.8 |  |  | **11** | **22** | **33** | 6.8 | 2.1 | 0.7 |
|  |  | **4** | **6** | 3.5 | 3.3 | 1.1 |  |  |  | **23** | **34** | 4.9 | 1.8 | 1.0 |
| **2** | **3** | **5** | **7** | 10.6 | 3.4 | 1.0 |  |  | **12** | **24** | **35** | 4.0 | 1.9 | 0.8 |
|  |  |  | **8** | 9.2 | 4.1 | 1.2 |  |  |  |  | **36** | 4.0 | 1.3 | 0.8 |
|  |  | **6** | **9** | 10.2 | 3.4 | 0.9 |  | **6** | **13** | **25** | **37** | 3.0 | 1.0 | 0.8 |
|  | **4** | **7** | **10** | 10.1 | 4.0 | 1.1 |  |  |  |  | **38** | 2.5 | 1.2 | 0.7 |
|  |  | **8** | **11** | 7.0 | 1.4 | 0.7 |  |  |  | **26** | **39** | 3.1 | 1.4 | 0.8 |
|  |  |  | **12** | 6.7 | 1.4 | 0.9 |  |  |  | **27** | **40** | 3.2 | 0.9 | 0.8 |
| **3** | **5** | **9** | **13** | 7.2 | 3.7 | 1.5 |  |  |  |  | **41** | 2.5 | 0.5 | 0.6 |
|  |  |  | **14** | 6.5 | 3.2 | 1.3 |  |  | **14** | **28** | **42** | 3.9 | 1.9 | 1.3 |
|  |  | **10** | **15** | 9.3 | 3.3 | 1.1 |  |  |  | **29** | **43** | 3.9 | 2.7 | 1.2 |
|  | **6** | **11** | **16** | 3.7 | 1.9 | 0.8 |  |  |  | **30** | **44** | 2.4 | 0.5 | 0.6 |
|  |  |  | **17** | 4.8 | 2.6 | 0.7 |  |  |  |  | **45** | 2.9 | 0.6 | 0.9 |
|  |  | **12** | **18** | 3.8 | 3.0 | 1.6 |  | **7** | **15** | **31** | **46** | 3.5 | 2.6 | 1.8 |
|  |  |  | **19** | 4.6 | 2.2 | 1.2 |  |  |  |  | **47** | 3.3 | 3.1 | 3.2 |
|  |  | **13** | **20** | 6.1 | 4.1 | 1.1 |  |  |  |  | **48** | 3.2 | 1.7 | 1.4 |
|  |  |  | **21** | 5.7 | 3.9 | 1.2 |  |  |  | **32** | **49** | 2.7 | 1.0 | 1.0 |
| **4** | **7** | **14** | **22** | 5.0 | 1.3 | 0.7 |  |  |  |  | **50** | 2.7 | 1.4 | 1.3 |
|  | **8** | **15** | **23** | 6.6 | 2.0 | 1.1 |  | **8** | **16** | **33** | **51** | 3.6 | 3.3 | 1.9 |
|  |  | **16** | **24** | 10.6 | 1.7 | 1.1 |  |  |  | **34** | **52** | 2.7 | 1.4 | 1.2 |
|  |  |  | **25** | 7.1 | 2.0 | 0.7 |  |  |  | **35** | **53** | 2.6 | 1.3 | 1.2 |
|  |  | **17** | **26** | 11.6 | 4.5 | 1.9 |  |  |  | **36** | **54** | 2.9 | 1.8 | 1.6 |
|  | **9** | **18** | **27** | 4.4 | 2.8 | 1.3 |  |  |  |  | **55** | 2.5 | 2.2 | 2.5 |
|  |  | **19** | **28** | 4.4 | 3.5 | 2.1 |  |  |  | **37** | **56** | 2.7 | 1.2 | 1.1 |
|  |  |  |  |  |  |  |  |  | **17** | **38** | **57** | 2.5 | 0.5 | 0.8 |
|  |  |  |  |  |  |  |  |  |  | **39** | **58** | 2.7 | 1.0 | 1.1 |
|  |  |  |  |  |  |  |  |  |  | **40** | **59** | 2.8 | 0.8 | 0.9 |

**Sources**: Occupations are clustered using Ward hierarchical method based on 72 principal components derived from 220 occupational descriptors from O*NET. For each occupation descriptors related to education, experience, and job training are aggregated into 3 corresponding variables, and then averaged across occupations in each group. Own calculations.

**S1 Table TA4. The Average Scores for Work Values and Interests**

| **Classification** | | | | **Work Values** | | | | | | **Work Interests** | | | | | |
| --- | --- | --- | --- | --- | --- | --- | --- | --- | --- | --- | --- | --- | --- | --- | --- |
| **Cluster** | **Group** | **Sub-group** | **Micro-group** | **Achievement** | **Independence** | **Recognition** | **Relationships** | **Support** | **Working Cond** | **Artistic** | **Conventional** | **Enterprising** | **Investigative** | **Realistic** | **Social** |
| **1** | **1** | **1** | **1** | 9 | 10 | 9 | 10 | 7 | 9 | 4 | 6 | 10 | 3 | 2 | 8 |
|  |  |  | **2** | 8 | 9 | 7 | 7 | 8 | 9 | 3 | 7 | 10 | 4 | 4 | 4 |
|  |  |  | **3** | 9 | 9 | 8 | 9 | 7 | 9 | 5 | 6 | 9 | 5 | 2 | 7 |
|  |  | **2** | **4** | 9 | 8 | 7 | 6 | 5 | 8 | 9 | 4 | 6 | 6 | 6 | 3 |
|  | **2** | **3** | **5** | 8 | 9 | 7 | 10 | 7 | 8 | 3 | 7 | 10 | 3 | 3 | 7 |
|  |  | **4** | **6** | 7 | 9 | 7 | 8 | 7 | 7 | 2 | 7 | 10 | 3 | 7 | 4 |
| **2** | **3** | **5** | **7** | 9 | 9 | 9 | 7 | 5 | 9 | 6 | 4 | 4 | 9 | 6 | 9 |
|  |  |  | **8** | 9 | 9 | 8 | 9 | 5 | 8 | 6 | 5 | 4 | 7 | 4 | 9 |
|  |  | **6** | **9** | 9 | 9 | 9 | 8 | 5 | 9 | 6 | 4 | 4 | 8 | 2 | 10 |
|  | **4** | **7** | **10** | 10 | 10 | 9 | 10 | 6 | 9 | 5 | 4 | 5 | 8 | 3 | 9 |
|  |  | **8** | **11** | 8 | 8 | 7 | 10 | 7 | 7 | 5 | 5 | 5 | 6 | 2 | 10 |
|  |  |  | **12** | 9 | 8 | 7 | 11 | 7 | 8 | 7 | 5 | 5 | 5 | 3 | 10 |
| **3** | **5** | **9** | **13** | 9 | 9 | 9 | 6 | 8 | 9 | 4 | 6 | 6 | 9 | 8 | 2 |
|  |  |  | **14** | 8 | 7 | 7 | 7 | 7 | 8 | 3 | 6 | 4 | 9 | 8 | 3 |
|  |  | **10** | **15** | 9 | 9 | 9 | 6 | 6 | 8 | 5 | 5 | 3 | 10 | 7 | 4 |
|  | **6** | **11** | **16** | 7 | 7 | 6 | 6 | 8 | 6 | 2 | 8 | 6 | 5 | 7 | 4 |
|  |  |  | **17** | 7 | 8 | 6 | 7 | 6 | 7 | 7 | 6 | 4 | 5 | 8 | 3 |
|  |  | **12** | **18** | 6 | 7 | 6 | 6 | 8 | 7 | 3 | 7 | 3 | 8 | 10 | 2 |
|  |  |  | **19** | 6 | 7 | 6 | 6 | 8 | 6 | 2 | 7 | 4 | 7 | 9 | 2 |
|  |  | **13** | **20** | 8 | 8 | 8 | 6 | 8 | 9 | 4 | 8 | 5 | 8 | 7 | 3 |
|  |  |  | **21** | 8 | 8 | 8 | 7 | 8 | 8 | 5 | 6 | 4 | 8 | 8 | 2 |
| **4** | **7** | **14** | **22** | 7 | 7 | 6 | 9 | 9 | 7 | 2 | 6 | 4 | 6 | 8 | 7 |
|  | **8** | **15** | **23** | 9 | 9 | 7 | 10 | 7 | 8 | 4 | 4 | 5 | 5 | 6 | 10 |
|  |  | **16** | **24** | 9 | 9 | 9 | 10 | 7 | 9 | 3 | 4 | 3 | 10 | 7 | 8 |
|  |  |  | **25** | 9 | 8 | 7 | 10 | 8 | 8 | 3 | 4 | 3 | 7 | 6 | 10 |
|  |  | **17** | **26** | 10 | 10 | 10 | 10 | 8 | 9 | 3 | 4 | 4 | 10 | 8 | 8 |
|  | **9** | **18** | **27** | 8 | 8 | 7 | 8 | 8 | 7 | 2 | 6 | 7 | 5 | 9 | 5 |
|  |  | **19** | **28** | 8 | 8 | 8 | 7 | 10 | 8 | 2 | 7 | 6 | 5 | 9 | 3 |

**S1 Table TA4. The Average Scores for Work Values and Interests (continued)**

| **Classification** | | | | **Work Values** | | | | | | **Work Interests** | | | | | |
| --- | --- | --- | --- | --- | --- | --- | --- | --- | --- | --- | --- | --- | --- | --- | --- |
| **Cluster** | **Group** | **Sub-group** | **Micro-group** | **Achievement** | **Independence** | **Recognition** | **Relationships** | **Support** | **Working Cond** | **Artistic** | **Conventional** | **Enterprising** | **Investigative** | **Realistic** | **Social** |
| **5** | **10** | **20** | **29** | 8 | 8 | 7 | 7 | 7 | 8 | 2 | 8 | 8 | 6 | 3 | 3 |
|  |  |  | **30** | 9 | 9 | 8 | 4 | 5 | 8 | 4 | 8 | 5 | 10 | 3 | 2 |
|  |  | **21** | **31** | 7 | 7 | 7 | 8 | 7 | 7 | 2 | 9 | 8 | 4 | 3 | 4 |
|  |  |  | **32** | 8 | 8 | 7 | 8 | 6 | 7 | 3 | 8 | 10 | 3 | 4 | 5 |
|  | **11** | **22** | **33** | 8 | 8 | 7 | 7 | 6 | 7 | 6 | 6 | 6 | 6 | 3 | 5 |
|  |  | **23** | **34** | 7 | 7 | 6 | 9 | 5 | 6 | 8 | 4 | 6 | 3 | 3 | 8 |
|  | **12** | **24** | **35** | 5 | 6 | 5 | 8 | 7 | 6 | 2 | 10 | 7 | 3 | 3 | 5 |
|  |  |  | **36** | 5 | 5 | 4 | 7 | 7 | 5 | 3 | 10 | 5 | 3 | 4 | 4 |
| **6** | **13** | **25** | **37** | 6 | 7 | 5 | 8 | 8 | 6 | 2 | 6 | 6 | 3 | 8 | 6 |
|  |  |  | **38** | 5 | 6 | 4 | 7 | 6 | 4 | 3 | 6 | 6 | 3 | 8 | 4 |
|  |  | **26** | **39** | 5 | 6 | 5 | 8 | 7 | 5 | 2 | 10 | 7 | 2 | 5 | 5 |
|  |  | **27** | **40** | 5 | 5 | 4 | 8 | 7 | 5 | 2 | 7 | 5 | 3 | 8 | 6 |
|  |  |  | **41** | 3 | 5 | 3 | 7 | 6 | 4 | 2 | 7 | 7 | 2 | 7 | 5 |
|  | **14** | **28** | **42** | 7 | 7 | 5 | 7 | 4 | 6 | 9 | 4 | 6 | 3 | 7 | 4 |
|  |  | **29** | **43** | 8 | 7 | 7 | 9 | 5 | 6 | 6 | 3 | 6 | 3 | 8 | 7 |
|  |  | **30** | **44** | 4 | 5 | 4 | 8 | 6 | 4 | 2 | 8 | 8 | 2 | 5 | 5 |
|  |  |  | **45** | 5 | 5 | 5 | 9 | 4 | 4 | 5 | 5 | 8 | 2 | 8 | 5 |
| **7** | **15** | **31** | **46** | 6 | 6 | 5 | 6 | 8 | 6 | 2 | 6 | 3 | 4 | 10 | 2 |
|  |  |  | **47** | 6 | 7 | 5 | 6 | 9 | 7 | 2 | 5 | 4 | 5 | 10 | 2 |
|  |  |  | **48** | 5 | 7 | 4 | 6 | 7 | 6 | 2 | 6 | 3 | 5 | 10 | 2 |
|  |  | **32** | **49** | 4 | 6 | 4 | 6 | 8 | 5 | 2 | 6 | 3 | 4 | 10 | 3 |
|  |  |  | **50** | 4 | 6 | 4 | 7 | 9 | 5 | 2 | 6 | 3 | 4 | 10 | 2 |
| **8** | **16** | **33** | **51** | 5 | 6 | 4 | 5 | 7 | 6 | 5 | 6 | 3 | 5 | 10 | 2 |
|  |  | **34** | **52** | 4 | 5 | 4 | 6 | 7 | 5 | 2 | 6 | 3 | 4 | 10 | 2 |
|  |  | **35** | **53** | 4 | 5 | 3 | 6 | 7 | 4 | 2 | 5 | 3 | 3 | 10 | 2 |
|  |  | **36** | **54** | 4 | 5 | 3 | 5 | 6 | 5 | 2 | 5 | 3 | 3 | 10 | 2 |
|  |  |  | **55** | 4 | 6 | 4 | 6 | 7 | 5 | 3 | 5 | 3 | 3 | 10 | 2 |
|  |  | **37** | **56** | 4 | 5 | 3 | 6 | 8 | 4 | 2 | 6 | 3 | 4 | 10 | 2 |
|  | **17** | **38** | **57** | 3 | 4 | 3 | 6 | 6 | 4 | 2 | 7 | 4 | 2 | 8 | 5 |
|  |  | **39** | **58** | 3 | 4 | 3 | 6 | 7 | 4 | 2 | 6 | 3 | 3 | 10 | 2 |
|  |  | **40** | **59** | 4 | 6 | 3 | 6 | 6 | 5 | 2 | 7 | 5 | 2 | 9 | 3 |

**Sources**: Occupations are clustered using Ward hierarchical method based on 72 principal components derived from 220 occupational descriptors from O*NET. Descriptors related to Work Values and Work Interests are averaged across occupations in each group and re-scaled to be in [1-10] interval. Own calculations.

**S1 Table TA5. Summary of OES Annual Average Wages**

| **Classification** | | | | **Mean annual wage (1000 USD)** | **St.Dev in annual wage (1000 USD)** | **Coeff of var (%)** |  | **Classification** | | | | **Mean annual wage (1000 USD)** | **St.Dev in annual wage (1000 USD)** | **Coeff of var (%)** |
| --- | --- | --- | --- | --- | --- | --- | --- | --- | --- | --- | --- | --- | --- | --- |
| **Cluster** | **Group** | **Sub-group** | **Micro-group** |  |  |  |  | **Cluster** | **Group** | **Sub-group** | **Micro-group** |  |  |  |
| **1** | **1** | **1** | **1** | 116.4 | 39.9 | 34 |  | **5** | **10** | **20** | **29** | 94.4 | 27.6 | 29 |
|  |  |  | **2** | 111.0 | 31.5 | 28 |  |  |  |  | **30** | 102.5 | 11.7 | 11 |
|  |  |  | **3** | 86.8 | 33.9 | 39 |  |  |  | **21** | **31** | 67.8 | 15.5 | 23 |
|  |  | **2** | **4** | 77.4 | 18.3 | 24 |  |  |  |  | **32** | 69.2 | 15.2 | 22 |
|  | **2** | **3** | **5** | 71.7 | 30.0 | 42 |  |  | **11** | **22** | **33** | 61.4 | 9.6 | 16 |
|  |  | **4** | **6** | 62.2 | 19.9 | 32 |  |  |  | **23** | **34** | 49.7 | 17.8 | 36 |
| **2** | **3** | **5** | **7** | 101.7 | 10.7 | 11 |  |  | **12** | **24** | **35** | 43.5 | 7.6 | 17 |
|  |  |  | **8** | 83.6 | 17.2 | 21 |  |  |  |  | **36** | 37.8 | 3.8 | 10 |
|  |  | **6** | **9** | 92.6 | 16.5 | 18 |  | **6** | **13** | **25** | **37** | 41.9 | 13.2 | 32 |
|  | **4** | **7** | **10** | 118.2 | 60.5 | 51 |  |  |  |  | **38** | 34.3 | 12.5 | 37 |
|  |  | **8** | **11** | 58.7 | 16.6 | 28 |  |  |  | **26** | **39** | 40.3 | 8.5 | 21 |
|  |  |  | **12** | 64.2 | 2.2 | 3 |  |  |  | **27** | **40** | 32.7 | 6.8 | 21 |
| **3** | **5** | **9** | **13** | 110.8 | 21.3 | 19 |  |  |  |  | **41** | 24.8 | 1.3 | 5 |
|  |  |  | **14** | 76.0 | 16.6 | 22 |  |  | **14** | **28** | **42** | 48.3 | 17.0 | 35 |
|  |  | **10** | **15** | 89.2 | 20.8 | 23 |  |  |  | **29** | **43** | 57.3 | 20.3 | 35 |
|  | **6** | **11** | **16** | 59.3 | 17.8 | 30 |  |  |  | **30** | **44** | 30.1 | 5.0 | 17 |
|  |  |  | **17** | 67.0 | 16.7 | 25 |  |  |  |  | **45** | 32.1 | 6.0 | 19 |
|  |  | **12** | **18** | 66.0 | 8.5 | 13 |  | **7** | **15** | **31** | **46** | 59.0 | 10.9 | 18 |
|  |  |  | **19** | 57.9 | 14.8 | 26 |  |  |  |  | **47** | 61.5 | 12.4 | 20 |
|  |  | **13** | **20** | 94.5 | 20.5 | 22 |  |  |  |  | **48** | 46.1 | 10.4 | 23 |
|  |  |  | **21** | 85.7 | 23.6 | 28 |  |  |  | **32** | **49** | 43.3 | 9.1 | 21 |
| **4** | **7** | **14** | **22** | 58.4 | 17.1 | 29 |  |  |  |  | **50** | 56.5 | 8.3 | 15 |
|  | **8** | **15** | **23** | 62.6 | 11.1 | 18 |  | **8** | **16** | **33** | **51** | 47.0 | 9.0 | 19 |
|  |  | **16** | **24** | 112.8 | 33.6 | 30 |  |  |  | **34** | **52** | 42.0 | 6.9 | 16 |
|  |  |  | **25** | 93.7 | 44.6 | 48 |  |  |  | **35** | **53** | 40.3 | 9.6 | 24 |
|  |  | **17** | **26** | 220.5 | 35.3 | 16 |  |  |  | **36** | **54** | 43.0 | 5.7 | 13 |
|  | **9** | **18** | **27** | 61.4 | 15.8 | 26 |  |  |  |  | **55** | 48.8 | 7.3 | 15 |
|  |  | **19** | **28** | 111.5 | 33.7 | 30 |  |  |  | **37** | **56** | 38.6 | 5.6 | 15 |
|  |  |  |  |  |  |  |  |  | **17** | **38** | **57** | 30.7 | 7.0 | 23 |
|  |  |  |  |  |  |  |  |  |  | **39** | **58** | 32.8 | 8.8 | 27 |
|  |  |  |  |  |  |  |  |  |  | **40** | **59** | 37.8 | 7.6 | 20 |

**Sources**: Wage data are from the Occupation Employment Statistics survey (BLS); occupations are clustered using Ward hierarchical method based on 72 principal components derived from 220 occupational descriptors from O*NET. Own calculations.

**S1 Table TA6. Job Gains and Losses (in thousands) between 2019 and 2029**

| **Classification** | | | | **Jobs in 2019** | **Shr. in tot emp in 2019 (%)** | **Jobs lost by 2029** | **Jobs gained by 2029** | **Net chng. in jobs by 2029** |  | **Classification** | | | | **Jobs in 2019** | **Shr. in tot emp in 2019 (%)** | **Jobs lost by 2029** | **Jobs gained by 2029** | **Net chng. in jobs by 2029** |
| --- | --- | --- | --- | --- | --- | --- | --- | --- | --- | --- | --- | --- | --- | --- | --- | --- | --- | --- |
| **Cluster** | **Group** | **Sub-group** | **Micro-group** |  |  |  |  |  |  | **Cluster** | **Group** | **Sub-group** | **Micro-group** |  |  |  |  |  |
| **1** | **1** | **1** | **1** | 1155 | 2.2 | -39 | 30 | -9 |  | **5** | **10** | **20** | **29** | 5154 | 9.8 | -12 | 246 | 233 |
|  |  |  | **2** | 5500 | 10.5 | -186 | 73 | -113 |  |  |  |  | **30** | 171 | 0.3 | 0 | 38 | 38 |
|  |  |  | **3** | 3098 | 5.9 | -11 | 110 | 99 |  |  |  | **21** | **31** | 2899 | 5.5 | -62 | 36 | -26 |
|  |  | **2** | **4** | 464 | 0.9 | -24 | 3 | -21 |  |  |  |  | **32** | 2920 | 5.6 | -83 | 10 | -73 |
|  | **2** | **3** | **5** | 6192 | 11.8 | -118 | 263 | 145 |  |  | **11** | **22** | **33** | 1307 | 2.5 | -54 | 20 | -33 |
|  |  | **4** | **6** | 6905 | 13.2 | -283 | 64 | -219 |  |  |  | **23** | **34** | 1577 | 3.0 | -14 | 40 | 27 |
| **2** | **3** | **5** | **7** | 178 | 0.3 | 0 | 6 | 6 |  |  | **12** | **24** | **35** | 14831 | 28.3 | -1214 | 61 | -1153 |
|  |  |  | **8** | 821 | 1.6 | -1 | 64 | 63 |  |  |  |  | **36** | 495 | 0.9 | -101 | 0 | -101 |
|  |  | **6** | **9** | 544 | 1.0 | -3 | 13 | 10 |  | **6** | **13** | **25** | **37** | 6700 | 12.8 | -93 | 1072 | 979 |
|  | **4** | **7** | **10** | 841 | 1.6 | -4 | 33 | 29 |  |  |  |  | **38** | 2595 | 4.9 | -15 | 293 | 278 |
|  |  | **8** | **11** | 2235 | 4.3 | -2 | 256 | 255 |  |  |  | **26** | **39** | 2610 | 5.0 | -180 | 10 | -170 |
|  |  |  | **12** | 3485 | 6.6 | -5 | 1 | -4 |  |  |  | **27** | **40** | 4003 | 7.6 | -114 | 164 | 50 |
| **3** | **5** | **9** | **13** | 1241 | 2.4 | -13 | 23 | 10 |  |  |  |  | **41** | 3184 | 6.1 | -150 | 56 | -95 |
|  |  |  | **14** | 459 | 0.9 | -2 | 6 | 4 |  |  | **14** | **28** | **42** | 441 | 0.8 | -39 | 0 | -38 |
|  |  | **10** | **15** | 272 | 0.5 | -2 | 5 | 3 |  |  |  | **29** | **43** | 571 | 1.1 | 0 | 73 | 73 |
|  | **6** | **11** | **16** | 607 | 1.2 | -3 | 8 | 5 |  |  |  | **30** | **44** | 15303 | 29.2 | -633 | 312 | -321 |
|  |  |  | **17** | 276 | 0.5 | 0 | 18 | 18 |  |  |  |  | **45** | 976 | 1.9 | -39 | 38 | -1 |
|  |  | **12** | **18** | 390 | 0.7 | -7 | 1 | -5 |  | **7** | **15** | **31** | **46** | 3864 | 7.4 | -98 | 62 | -36 |
|  |  |  | **19** | 914 | 1.7 | -4 | 19 | 14 |  |  |  |  | **47** | 1521 | 2.9 | -5 | 43 | 39 |
|  |  | **13** | **20** | 4202 | 8.0 | -28 | 379 | 351 |  |  |  |  | **48** | 1066 | 2.0 | -41 | 12 | -29 |
|  |  |  | **21** | 1639 | 3.1 | -29 | 22 | -8 |  |  |  | **32** | **49** | 655 | 1.2 | -32 | 20 | -11 |
| **4** | **7** | **14** | **22** | 2590 | 4.9 | 0 | 221 | 220 |  |  |  |  | **50** | 2282 | 4.3 | -57 | 4 | -52 |
|  | **8** | **15** | **23** | 1022 | 1.9 | -6 | 100 | 94 |  | **8** | **16** | **33** | **51** | 258 | 0.5 | -26 | 3 | -23 |
|  |  | **16** | **24** | 702 | 1.3 | -26 | 13 | -13 |  |  |  | **34** | **52** | 4226 | 8.1 | -229 | 28 | -201 |
|  |  |  | **25** | 4299 | 8.2 | 0 | 295 | 295 |  |  |  | **35** | **53** | 2143 | 4.1 | -38 | 32 | -6 |
|  |  | **17** | **26** | 597 | 1.1 | -7 | 2 | -5 |  |  |  | **36** | **54** | 695 | 1.3 | -32 | 3 | -29 |
|  | **9** | **18** | **27** | 1644 | 3.1 | -7 | 34 | 26 |  |  |  |  | **55** | 2318 | 4.4 | -73 | 6 | -67 |
|  |  | **19** | **28** | 202 | 0.4 | -7 | 2 | -4 |  |  |  | **37** | **56** | 3482 | 6.6 | -211 | 11 | -199 |
|  |  |  |  |  |  |  |  |  |  |  | **17** | **38** | **57** | 2567 | 4.9 | -102 | 17 | -85 |
|  |  |  |  |  |  |  |  |  |  |  |  | **39** | **58** | 7866 | 15.0 | -39 | 109 | 70 |
|  |  |  |  |  |  |  |  |  |  |  |  | **40** | **59** | 3341 | 6.4 | -86 | 103 | 17 |

**Sources**: This table is based on BLS Employment Projections 2019-2029, Table 1.2 “Employment by detailed occupation, 2019 and projected 2029”. Occupations are clustered using Ward hierarchical method based on 72 principal components derived from 220 occupational descriptors from O*NET. Own calculations.

**S1 Table TA7. Reasons behind BLS Projections of Occupational Employment**

| **Classification** | | | | **Losses in jobs** | | | | | **Gains in jobs** | | | | |
| --- | --- | --- | --- | --- | --- | --- | --- | --- | --- | --- | --- | --- | --- |
| **Cluster** | **Group** | **Sub-group** | **Micro-group** | **Total job loss** | **Reason given** | **% with reason** | **Due to tech** | **% due to tech** | **Total job gain** | **Reason given** | **% with reason** | **Due to tech** | **% due to tech** |
| **1** | **1** | **1** | **1** | -39 | -39 | **100.0** | 0 | **0.0** | 30 | 0 | **0.0** | 0 | **0.0** |
|  |  |  | **2** | -186 | -57 | **30.4** | -56 | **30.3** | 73 | 0 | **0.0** | 0 | **0.0** |
|  |  |  | **3** | -11 | -10 | **93.9** | 0 | **0.0** | 110 | 83 | **75.2** | 0 | **0.0** |
|  |  | **2** | **4** | -24 | -2 | **10.1** | -2 | **10.1** | 3 | 3 | **100.0** | 0 | **0.0** |
|  | **2** | **3** | **5** | -118 | -108 | **91.0** | 0 | **0.0** | 263 | 128 | **48.5** | 10 | **3.8** |
|  |  | **4** | **6** | -283 | -37 | **13.1** | -26 | **9.2** | 64 | 11 | **17.9** | 0 | **0.0** |
| **2** | **3** | **5** | **7** | 0 | 0 | **0.0** | 0 | **0.0** | 6 | 6 | **96.2** | 0 | **0.0** |
|  |  |  | **8** | -1 | 0 | **0.0** | 0 | **0.0** | 64 | 55 | **86.8** | 0 | **0.0** |
|  |  | **6** | **9** | -3 | 0 | **0.0** | 0 | **0.0** | 13 | 9 | **72.1** | 0 | **0.0** |
|  | **4** | **7** | **10** | -4 | -2 | **44.0** | 0 | **0.0** | 33 | 17 | **51.7** | 0 | **0.0** |
|  |  | **8** | **11** | -2 | 0 | **0.0** | 0 | **0.0** | 256 | 102 | **39.8** | 1 | **0.2** |
|  |  |  | **12** | -5 | 0 | **0.0** | 0 | **0.0** | 1 | 0 | **0.0** | 0 | **0.0** |
| **3** | **5** | **9** | **13** | -13 | 0 | **2.1** | 0 | **0.0** | 23 | 23 | **98.3** | 0 | **0.8** |
|  |  |  | **14** | -2 | 0 | **0.0** | 0 | **0.0** | 6 | 0 | **0.0** | 0 | **0.0** |
|  |  | **10** | **15** | -2 | 0 | **0.0** | 0 | **0.0** | 5 | 1 | **12.6** | 1 | **12.6** |
|  | **6** | **11** | **16** | -3 | 0 | **0.0** | 0 | **0.0** | 8 | 0 | **0.0** | 0 | **0.0** |
|  |  |  | **17** | 0 | 0 | **0.0** | 0 | **0.0** | 18 | 18 | **96.7** | 8 | **43.2** |
|  |  | **12** | **18** | -7 | 0 | **0.0** | 0 | **0.0** | 1 | 0 | **27.3** | 0 | **27.3** |
|  |  |  | **19** | -4 | -1 | **30.0** | -1 | **30.0** | 19 | 3 | **14.1** | 3 | **14.1** |
|  |  | **13** | **20** | -28 | -28 | **100.0** | 0 | **0.0** | 379 | 362 | **95.5** | 332 | **87.8** |
|  |  |  | **21** | -29 | -15 | **50.9** | -8 | **28.9** | 22 | 22 | **99.7** | 22 | **99.7** |
| **4** | **7** | **14** | **22** | 0 | 0 | **100.0** | 0 | **0.0** | 221 | 159 | **72.0** | 10 | **4.4** |
|  | **8** | **15** | **23** | -6 | 0 | **0.0** | 0 | **0.0** | 100 | 30 | **30.0** | 0 | **0.0** |
|  |  | **16** | **24** | -26 | -22 | **87.1** | 0 | **0.0** | 13 | 0 | **0.0** | 0 | **0.0** |
|  |  |  | **25** | 0 | 0 | **0.0** | 0 | **0.0** | 295 | 253 | **85.7** | 4 | **1.5** |
|  |  | **17** | **26** | -7 | -7 | **95.8** | 0 | **0.0** | 2 | 0 | **0.0** | 0 | **0.0** |
|  | **9** | **18** | **27** | -7 | -6 | **87.5** | -1 | **8.3** | 34 | 0 | **1.2** | 0 | **0.0** |
|  |  | **19** | **28** | -7 | -2 | **31.0** | -2 | **31.0** | 2 | 0 | **0.0** | 0 | **0.0** |

**S1 Table TA7 (cont.). Reasons behind BLS Projections of Occupational Employment**

| **Classification** | | | | **Losses in jobs** | | | | | **Gains in jobs** | | | | |
| --- | --- | --- | --- | --- | --- | --- | --- | --- | --- | --- | --- | --- | --- |
| **Cluster** | **Group** | **Sub-group** | **Micro-group** | **Total job loss** | **Reason given** | **% with reason** | **Due to tech** | **% due to tech** | **Total job gain** | **Reason given** | **% with reason** | **Due to tech** | **% due to tech** |
| **5** | **10** | **20** | **29** | -12 | -12 | **100.00** | -12 | **100.00** | 246 | 216 | **87.82** | 103 | **41.93** |
|  |  |  | **30** | 0 | 0 | **0.00** | 0 | **0.00** | 38 | 38 | **100.00** | 38 | **100.00** |
|  |  | **21** | **31** | -62 | -58 | **92.74** | -53 | **85.60** | 36 | 25 | **68.27** | 25 | **68.27** |
|  |  |  | **32** | -83 | 0 | **0.00** | 0 | **0.00** | 10 | 0 | **0.00** | 0 | **0.00** |
|  | **11** | **22** | **33** | -54 | -4 | **6.75** | -4 | **6.75** | 20 | 0 | **0.00** | 0 | **0.00** |
|  |  | **23** | **34** | -14 | -1 | **8.47** | 0 | **0.00** | 40 | 13 | **31.25** | 0 | **0.00** |
|  | **12** | **24** | **35** | -1214 | -1214 | **100.00** | -1210 | **99.67** | 61 | 23 | **37.32** | 0 | **0.00** |
|  |  |  | **36** | -101 | -100 | **99.42** | -68 | **67.91** | 0 | 0 | **0.00** | 0 | **0.00** |
| **6** | **13** | **25** | **37** | -93 | -64 | **69.07** | -14 | **15.63** | 1072 | 1049 | **97.81** | 0 | **0.00** |
|  |  |  | **38** | -15 | -13 | **81.76** | 0 | **0.00** | 293 | 289 | **98.65** | 0 | **0.00** |
|  |  | **26** | **39** | -180 | -124 | **68.99** | -117 | **64.94** | 10 | 0 | **0.00** | 0 | **0.00** |
|  |  | **27** | **40** | -114 | -114 | **100.00** | -88 | **77.13** | 164 | 18 | **10.96** | 0 | **0.00** |
|  |  |  | **41** | -150 | -150 | **100.00** | 0 | **0.00** | 56 | 9 | **15.70** | 0 | **0.00** |
|  | **14** | **28** | **42** | -39 | 0 | **0.00** | 0 | **0.00** | 0 | 0 | **0.00** | 0 | **0.00** |
|  |  | **29** | **43** | 0 | 0 | **0.00** | 0 | **0.00** | 73 | 28 | **38.42** | 0 | **0.00** |
|  |  | **30** | **44** | -633 | -428 | **67.61** | -403 | **63.75** | 312 | 311 | **99.68** | 0 | **0.00** |
|  |  |  | **45** | -39 | -39 | **99.74** | 0 | **0.00** | 38 | 34 | **89.74** | 0 | **0.00** |
| **7** | **15** | **31** | **46** | -98 | -84 | **85.86** | -84 | **85.86** | 62 | 53 | **86.56** | 49 | **80.00** |
|  |  |  | **47** | -5 | 0 | **0.00** | 0 | **0.00** | 43 | 36 | **83.99** | 2 | **3.68** |
|  |  |  | **48** | -41 | -3 | **7.95** | -3 | **6.76** | 12 | 6 | **45.58** | 6 | **45.58** |
|  |  | **32** | **49** | -32 | -30 | **95.12** | -29 | **92.58** | 20 | 0 | **0.00** | 0 | **0.00** |
|  |  |  | **50** | -57 | -3 | **5.01** | -2 | **3.91** | 4 | 0 | **0.00** | 0 | **0.00** |
| **8** | **16** | **33** | **51** | -26 | -16 | **62.72** | -16 | **62.72** | 3 | 2 | **64.13** | 0 | **0.00** |
|  |  | **34** | **52** | -229 | -192 | **83.80** | -181 | **78.81** | 28 | 24 | **84.13** | 0 | **0.00** |
|  |  | **35** | **53** | -38 | -25 | **65.63** | -24 | **63.71** | 32 | 10 | **30.59** | 5 | **17.02** |
|  |  | **36** | **54** | -32 | -15 | **48.83** | -15 | **48.83** | 3 | 1 | **55.90** | 0 | **0.00** |
|  |  |  | **55** | -73 | -61 | **84.27** | 0 | **0.00** | 6 | 3 | **45.08** | 0 | **0.00** |
|  |  | **37** | **56** | -211 | -130 | **61.74** | -130 | **61.74** | 11 | 1 | **10.32** | 0 | **0.00** |
|  | **17** | **38** | **57** | -102 | -91 | **89.74** | -32 | **31.35** | 17 | 0 | **0.00** | 0 | **0.00** |
|  |  | **39** | **58** | -39 | -13 | **34.37** | -2 | **4.10** | 109 | 76 | **69.67** | 0 | **0.00** |
|  |  | **40** | **59** | -86 | -29 | **33.65** | -29 | **33.65** | 103 | 86 | **82.81** | 23 | **22.00** |

**Sources**: This table is based on BLS Employment Projections 2019-2029, Table 1.2 “Employment by detailed occupation, 2019 and projected 2029” and Table 1.12 “Factors affecting occupational utilization, projected 2019–29”. Occupations are clustered using Ward hierarchical method based on 72 principal components derived from 220 occupational descriptors from O*NET. Own calculations.
