# Supplementary material for: Assessing the impact of technological change on similar occupations: Implications for employment alternatives
Source: PLoS One. 2023 Sep 18;18(9):e0291428. doi: 10.1371/journal.pone.0291428 (PMC10506722; doi:10.1371/journal.pone.0291428)
Supplement: S2 Appendix — (DOCX) [file pone.0291428.s002.docx]

**S2 Appendices**

**Appendix A: Listing of Knowledge, Skills, and Abilities Reported by O*NET**

| **Knowledge** | **Skills** | **Abilities** | |
| --- | --- | --- | --- |
| Administration & Management | Active Learning | Arm-H& Steadiness | Response Orientation |
| Biology | Active Listening | Auditory Attention | Selective Attention |
| Building & Construction | Complex Problem Solving | Category Flexibility | Sound Localization |
| Chemistry | Coordination | Control Precision | Spatial Orientation |
| Clerical | Critical Thinking | Deductive Reasoning | Speech Clarity |
| Communications & Media | Equipment Maintenance | Depth Perception | Speech Recognition |
| Computers & Electronics | Equipment Selection | Dynamic Flexibility | Speed of Closure |
| Customer & Personal Service | Installation | Dynamic Strength | Speed of Limb Movement |
| Design | Instructing | Explosive Strength | Stamina |
| Economics & Accounting | Judgment & Decision Making | Extent Flexibility | Static Strength |
| Education & Training | Learning Strategies | Far Vision | Time Sharing |
| Engineering & Technology | Management of Financial Resources | Finger Dexterity | Trunk Strength |
| English Language | Management of Material Resources | Flexibility of Closure | Visual Color Discrimination |
| Fine Arts | Management of Personnel Resources | Fluency of Ideas | Visualization |
| Food Production | Mathematics | Glare Sensitivity | Wrist-Finger Speed |
| Foreign Language | Monitoring | Gross Body Coordination | Written Comprehension |
| Geography | Negotiation | Gross Body Equilibrium | Written Expression |
| History & Archeology | Operation Monitoring | Hearing Sensitivity |  |
| Law & Government | Operation & Control | Inductive Reasoning |  |
| Mathematics | Operations Analysis | Information Ordering |  |
| Mechanical | Persuasion | Manual Dexterity |  |
| Medicine & Dentistry | Programming | Mathematical Reasoning |  |
| Personnel & Human Resources | Quality Control Analysis | Memorization |  |
| Philosophy & Theology | Reading Comprehension | Multilimb Coordination |  |
| Physics | Repairing | Near Vision |  |
| Production & Processing | Science | Night Vision |  |
| Psychology | Service Orientation | Number Facility |  |
| Public Safety & Security | Social Perceptiveness | Oral Comprehension |  |
| Sales & Marketing | Speaking | Oral Expression |  |
| Sociology & Anthropology | Systems Analysis | Originality |  |
| Telecommunications | Systems Evaluation | Perceptual Speed |  |
| Therapy & Counseling | Technology Design | Peripheral Vision |  |
| Transportation | Time Management | Problem Sensitivity |  |
|  | Troubleshooting | Rate Control |  |
|  | Writing | Reaction Time |  |

**Sources**: Occupational descriptors from O*NET.

**S2 Appendix B. Listing of Education, Experience, and Training Categories from O*NET**

| **Education** | **Experience** | **Training** |
| --- | --- | --- |
| Below High School | None | None, demo |
| High School | (0,1] mo | (0,1] mo |
| Post-Sec | (1;3] mo | (1,3] mo |
| Some college | (3;6] mo | (3,6] mo |
| Assoc degree | (6;12] mo | (6,12] mo |
| BA degree | (1;2] yr | (1,2] yr |
| Post-BA cert | (2;4] yr | (2,4] yr |
| MA degree | (4;6] yr | (4,10] yr |
| Post-MA cert | (6;8] yr | 10+ yr |
| Prof degree | (8;10] yr |  |
| Doctorate | 10+ yr |  |
| Post-doc |  |  |

**Sources**: Occupational descriptors from O*NET.

**S2 Appendix C. Listing of Work activities, Values and Interests Reported by O*NET**

| **Work Activities** | | **Work Values** |
| --- | --- | --- |
| Analyzing Data or Information | Judging the Qualities of Things, Serv.. | Achievement |
| Assisting and Caring for Others | Making Decisions and Solving Problems | Independence |
| Coaching and Developing Others | Monitor Processes, Materials, or Surr.. | Recognition |
| Communicating with Persons Outside Or.. | Monitoring and Controlling Resources | Relationships |
| Communicating with Supervisors, Peers.. | Operating Vehicles, Mechanized Device.. | Support |
| Controlling Machines and Processes | Organizing, Planning, and Prioritizin.. | Working Conditions |
| Coordinating the Work and Activities .. | Performing Administrative Activities |  |
| Developing Objectives and Strategies | Performing General Physical Activities |  |
| Developing and Building Teams | Performing for or Working Directly wi.. |  |
| Documenting/Recording Information | Processing Information | **Work Interests** |
| Drafting, Laying Out, and Specifying .. | Provide Consultation and Advice to Ot.. | Artistic |
| Establishing and Maintaining Interper.. | Repairing and Maintaining Electronic .. | Conventional |
| Estimating the Quantifiable Character.. | Repairing and Maintaining Mechanical .. | Enterprising |
| Evaluating Information to Determine C.. | Resolving Conflicts and Negotiating w.. | Investigative |
| Getting Information | Scheduling Work and Activities | Realistic |
| Guiding, Directing, and Motivating Su.. | Selling or Influencing Others | Social |
| Handling and Moving Objects | Staffing Organizational Units |  |
| Identifying Objects, Actions, and Eve.. | Thinking Creatively |  |
| Inspecting Equipment, Structures, or .. | Training and Teaching Others |  |
| Interacting With Computers | Updating and Using Relevant Knowledge |  |
| Interpreting the Meaning of Informati.. |  |  |

**Sources**: Occupational descriptors from O*NET.

**S2 Appendix D. The Full Listing of Occupations and Their Taxonomy**

**
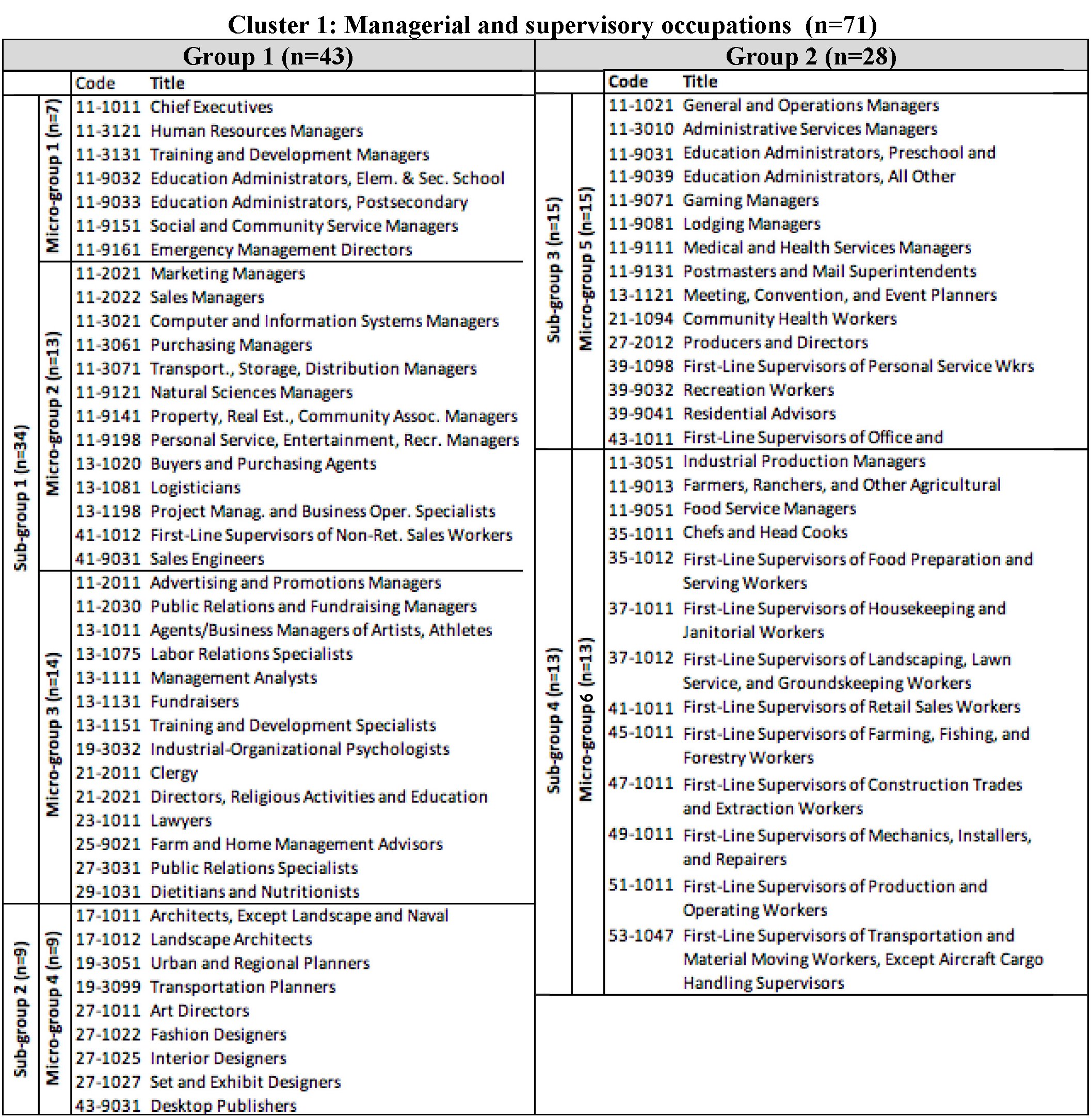
**


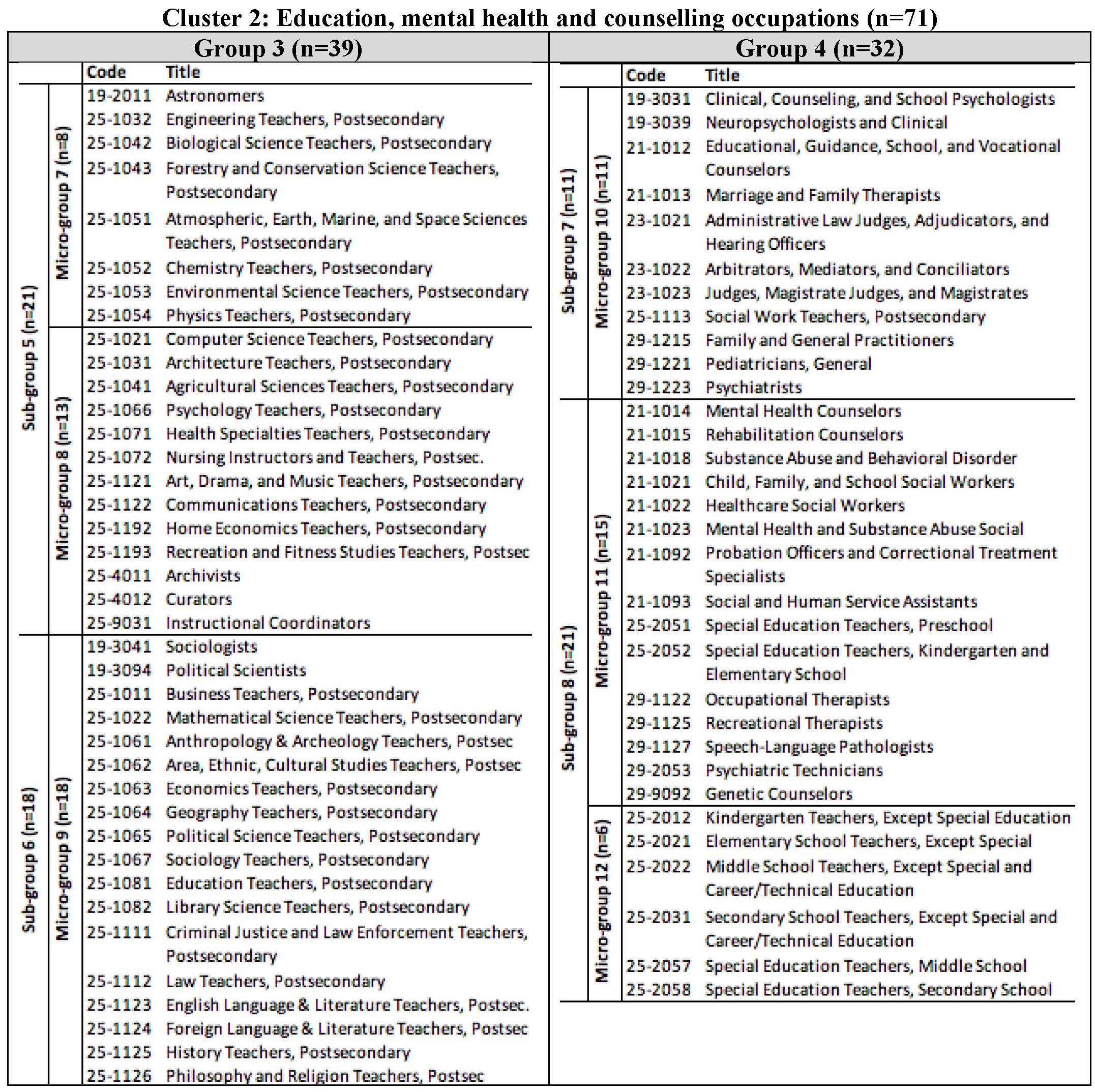


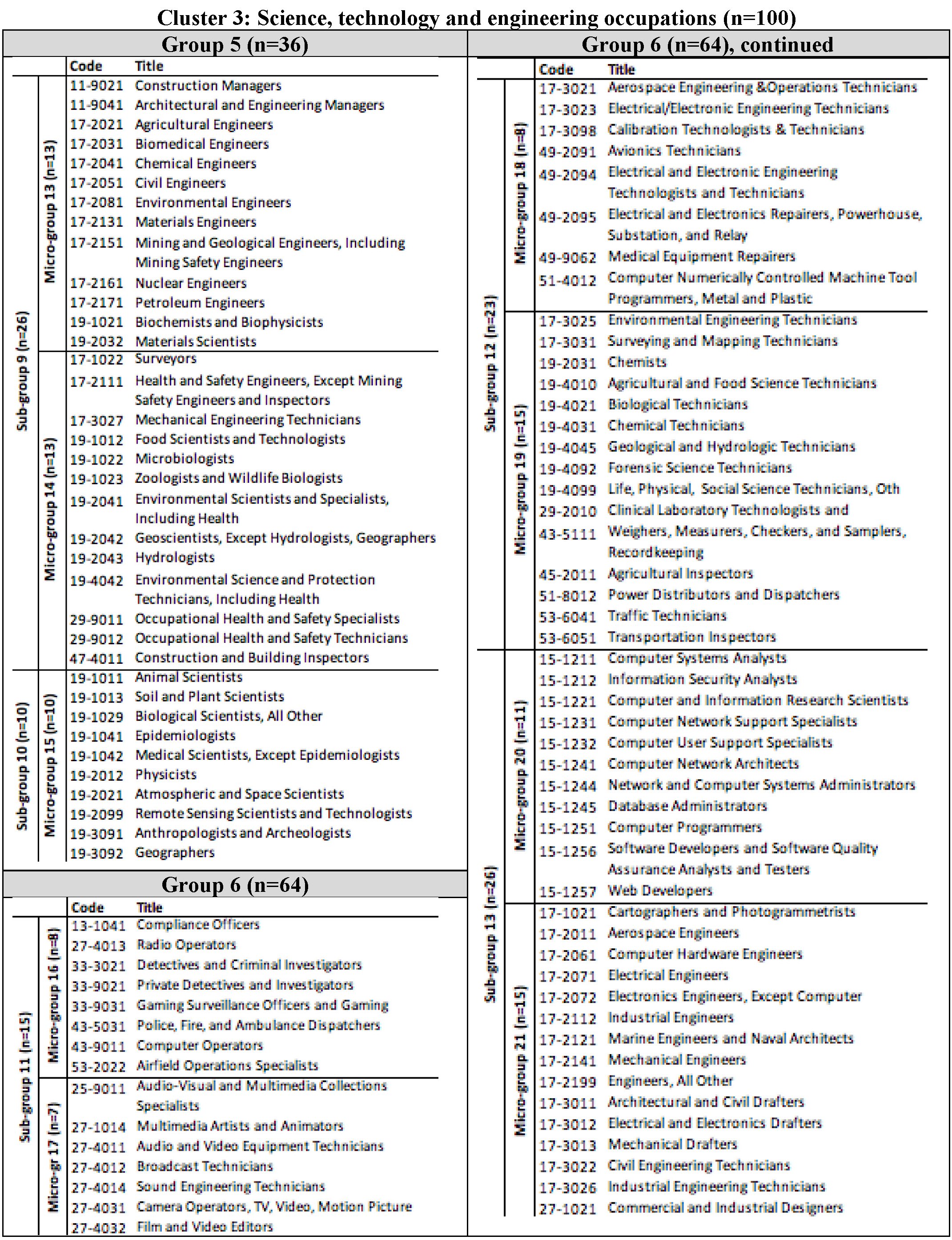


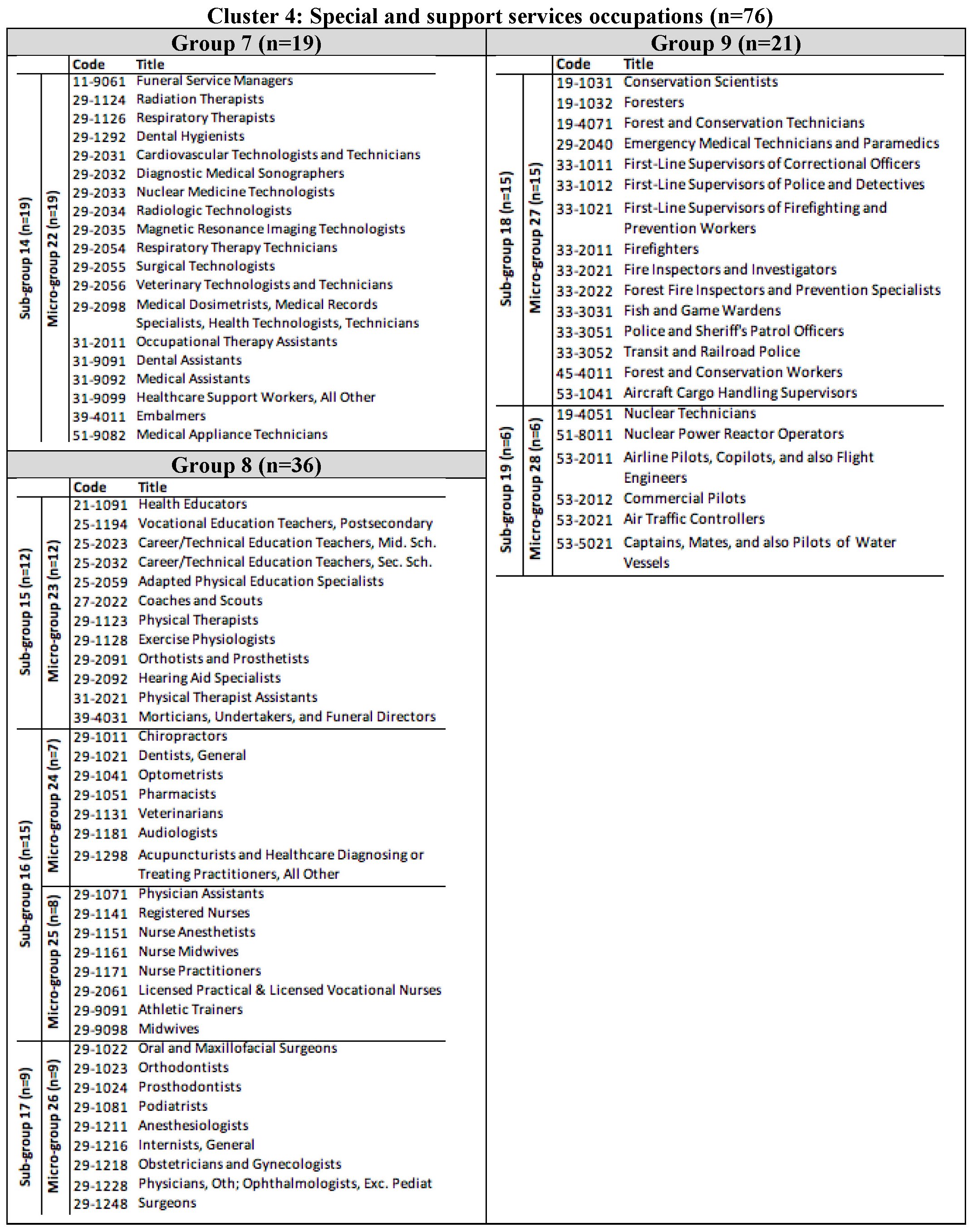


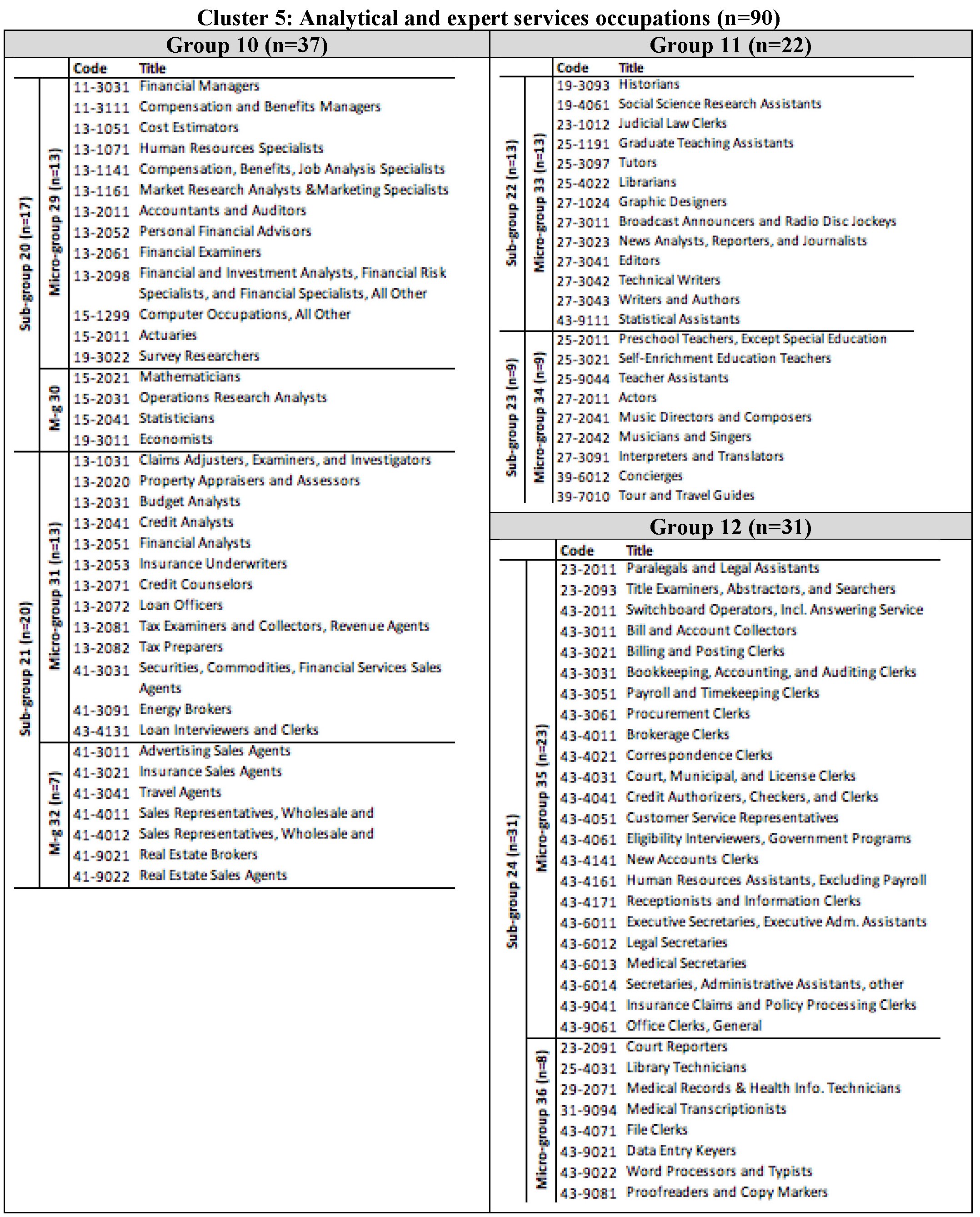


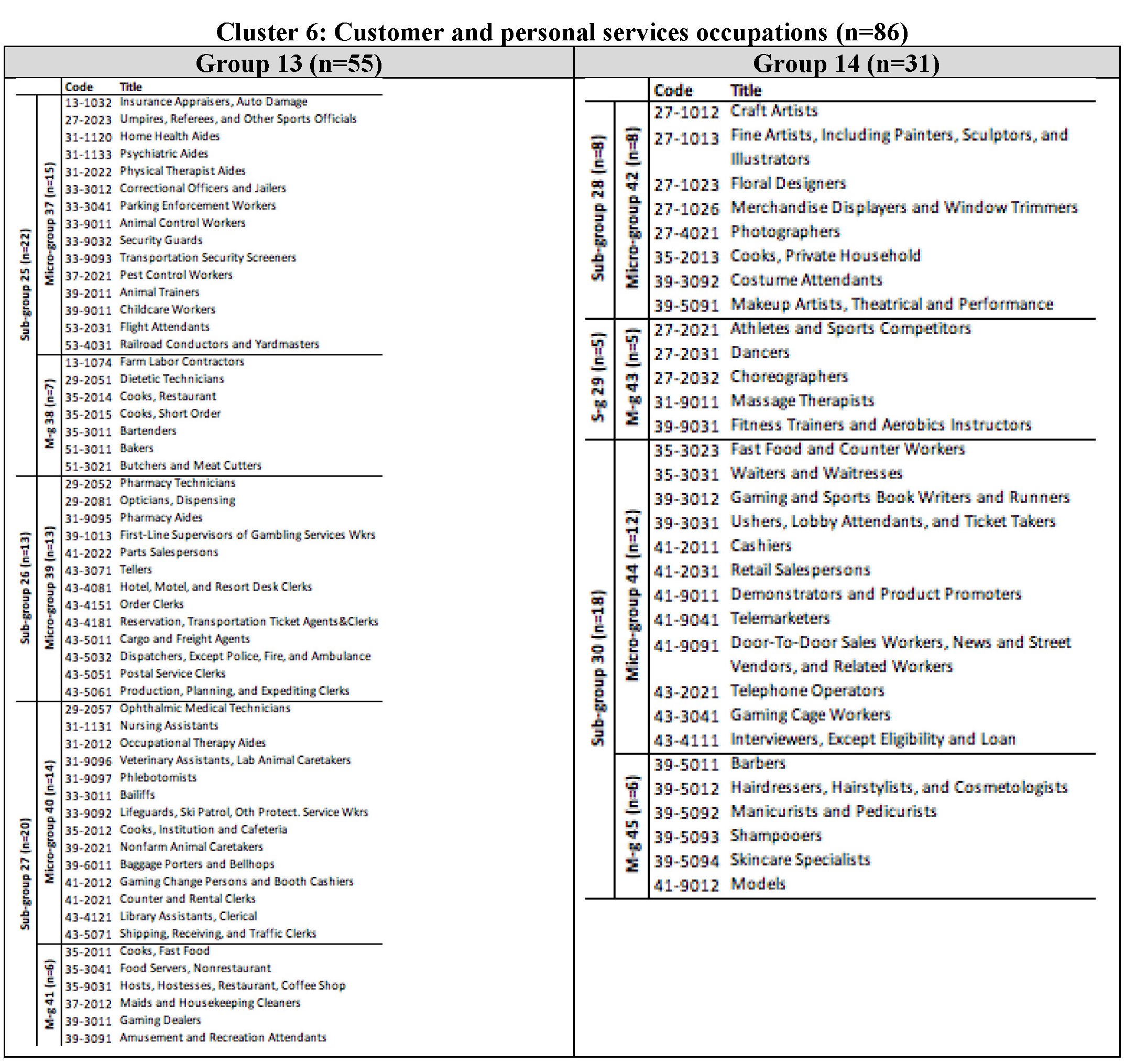


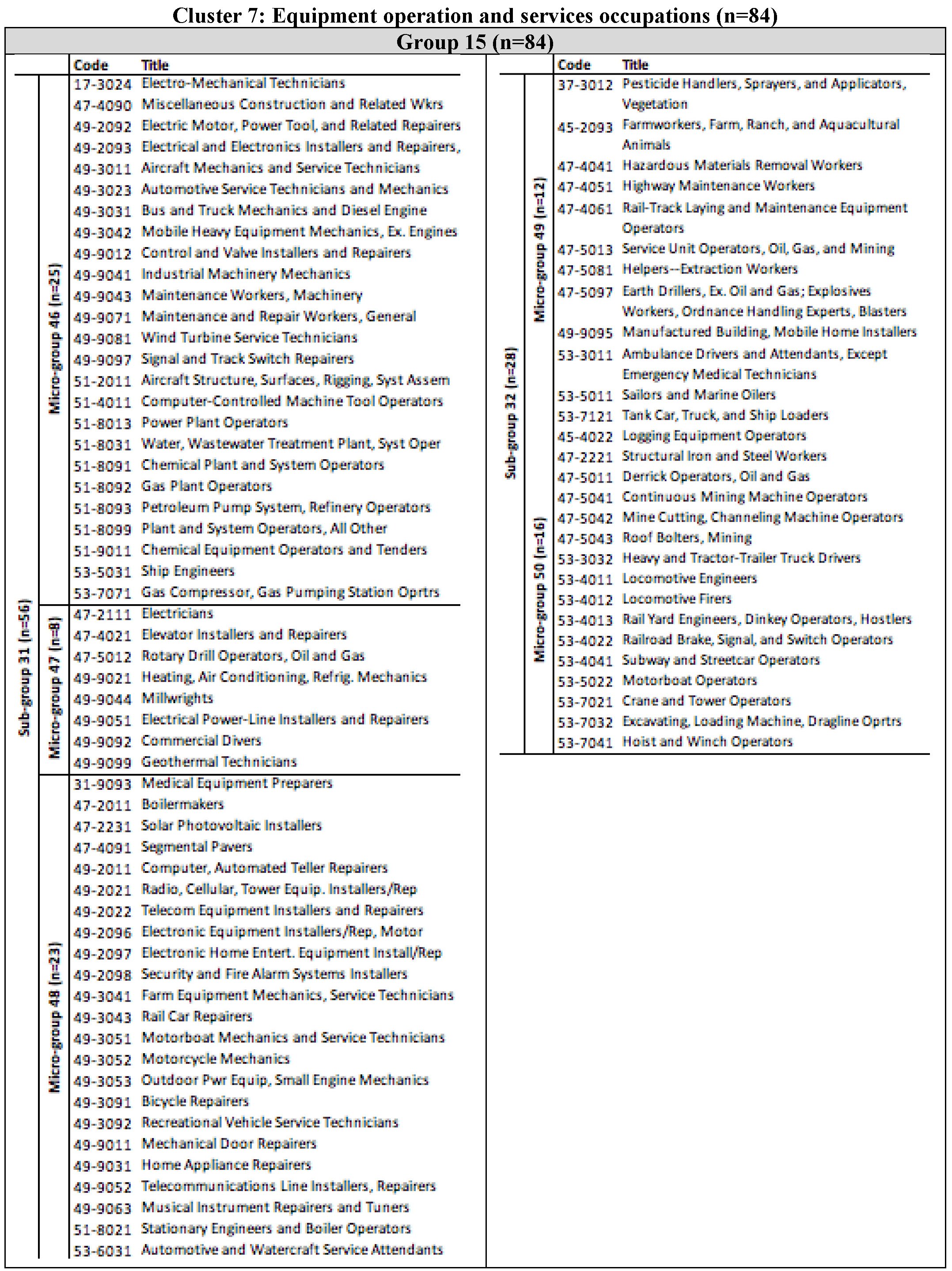


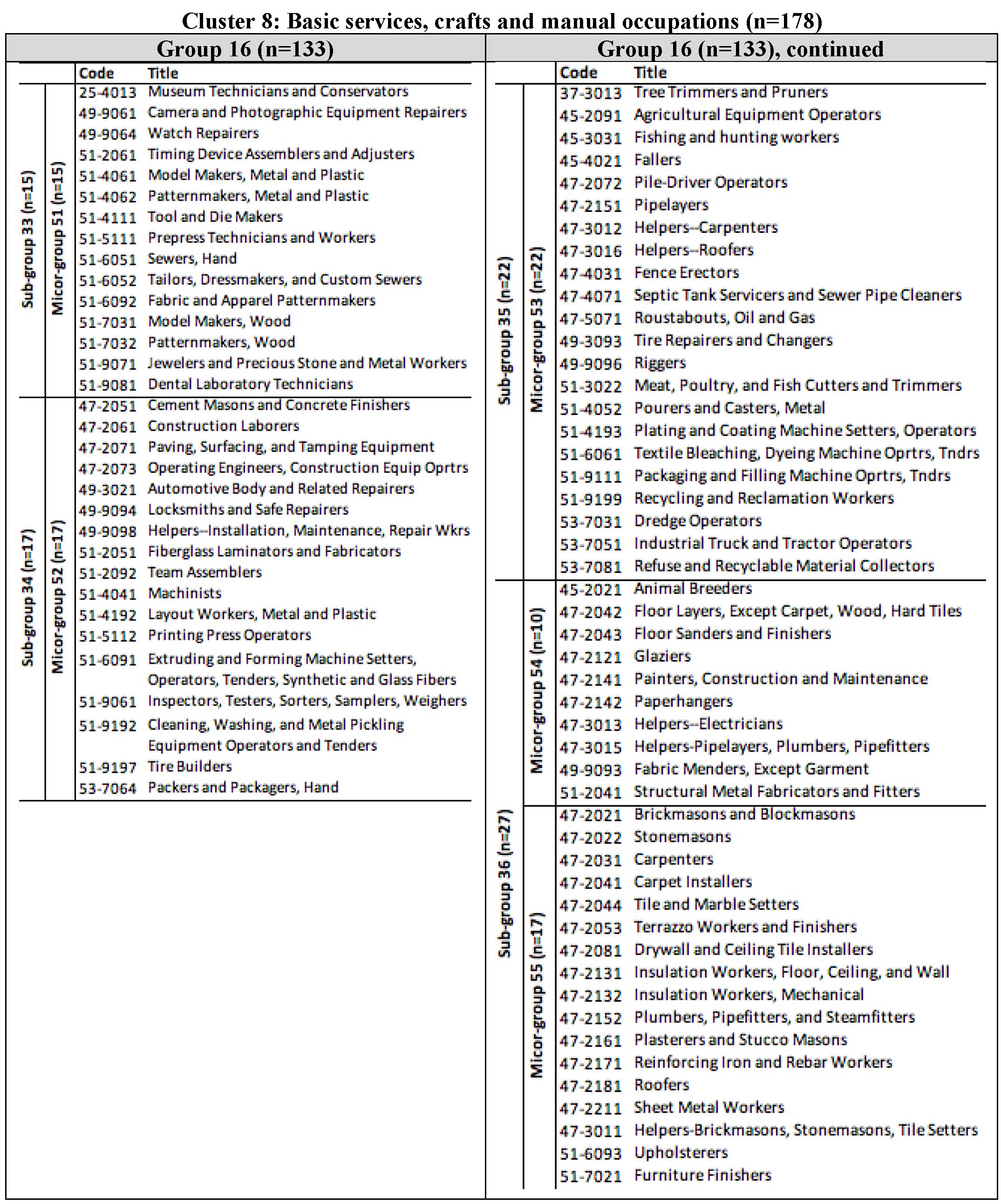


**S2 Appendix E.** Summary of OES Annual Average Wages for 17 Occupational Groups

| **Cluster** | **Group** | **Mean Annual Wage**  **(in 1000 USD)** | **Standard Deviation**  **(in 1000 USD)** | **Coefficient of Variation (in percent)** |
| --- | --- | --- | --- | --- |
| 1 | 1 | 97.0 | 34.2 | 35.3 |
|  | 2 | 67.3 | 25.8 | 38.4 |
| 2 | 3 | 91.5 | 16.7 | 18.3 |
|  | 4 | 80.9 | 46.2 | 57.1 |
| 3 | 5 | 93.2 | 24.3 | 26.1 |
|  | 6 | 73.6 | 22.9 | 31.2 |
| 4 | 7 | 58.4 | 17.1 | 29.2 |
|  | 8 | 118.7 | 69.3 | 58.4 |
|  | 9 | 75.7 | 31.6 | 41.7 |
| 5 | 10 | 81.5 | 24.4 | 29.9 |
|  | 11 | 56.4 | 14.6 | 25.8 |
|  | 12 | 42.3 | 7.3 | 17.3 |
| 6 | 13 | 36.3 | 11.0 | 30.3 |
|  | 14 | 39.5 | 16.0 | 40.6 |
| 7 | 15 | 52.9 | 12.2 | 23.1 |
| 8 | 16 | 41.9 | 8.0 | 19.1 |
|  | 17 | 33.8 | 8.3 | 24.4 |

**Sources**: Wage data are from the Occupation Employment Statistics survey (BLS); occupations are clustered using Ward hierarchical method based on 72 principal components derived from 220 occupational descriptors from O*NET. Own calculations.

**S2 Appendix F. Job Gains and Losses (in thousands) between 2019 and 2029, for 17 Groups**

| **Cluster** | **Group** | J**obs in 2019** | **Share in total employment in 2019, %** | **Jobs lost by 2029** | **Jobs gained by 2029** | **Net change in jobs by 2029** |
| --- | --- | --- | --- | --- | --- | --- |
| 1 | 1 | 10,216 | 6.6 | -260 | 216 | -44 |
|  | 2 | 13,097 | 8.5 | -401 | 327 | -74 |
| 2 | 3 | 1,543 | 1.0 | -4 | 83 | 78 |
|  | 4 | 6,561 | 4.2 | -10 | 290 | 280 |
| 3 | 5 | 1,971 | 1.3 | -17 | 34 | 17 |
|  | 6 | 8,028 | 5.2 | -72 | 446 | 375 |
| 4 | 7 | 2,590 | 1.7 | 0 | 221 | 220 |
|  | 8 | 6,619 | 4.3 | -39 | 410 | 372 |
|  | 9 | 1,846 | 1.2 | -14 | 36 | 22 |
| 5 | 10 | 11,144 | 7.2 | -158 | 329 | 171 |
|  | 11 | 2,884 | 1.9 | -67 | 61 | -6 |
|  | 12 | 15,326 | 9.9 | -1,315 | 61 | -1,254 |
| 6 | 13 | 19,091 | 12.4 | -552 | 1,595 | 1,042 |
|  | 14 | 17,290 | 11.2 | -710 | 424 | -286 |
| 7 | 15 | 9,388 | 6.1 | -232 | 142 | -90 |
| 8 | 16 | 13,122 | 8.5 | -609 | 83 | -525 |
|  | 17 | 13,774 | 8.9 | -227 | 229 | 2 |

**Sources**: This table is based on BLS Employment Projections 2019-2029, Table 1.2 “Employment by detailed occupation, 2019 and projected 2029”. Occupations are clustered using Ward hierarchical method based on 72 principal components derived from 220 occupational descriptors from O*NET. Own calculations.

**S2 Appendix G. Reasons behind BLS Projections of Occupational Employment, for 17 Groups**

| **Cluster** | **Group** | **Losses in jobs** | | | | | **Gains in jobs** | | | | |
| --- | --- | --- | --- | --- | --- | --- | --- | --- | --- | --- | --- |
|  |  | **Total**  **job losses** | **Reason given** | **% with reason** | **Due to tech** | **% due to tech** | **Total**  **job**  **gains** | **Reason given** | **% with reason** | **Due to tech** | **% due to tech** |
| 1 | 1 | -260 | -109 | **41.8** | -59 | **22.5** | 216 | 86 | **39.7** | 0 | **0.0** |
|  | 2 | -401 | -145 | **36.1** | -26 | **6.5** | 327 | 139 | **42.5** | 10 | **3.1** |
| 2 | 3 | -4 | 0 | **0.0** | 0 | **0.0** | 83 | 70 | **85.2** | 0 | **0.0** |
|  | 4 | -10 | -2 | **16.6** | 0 | **0.0** | 290 | 119 | **41.0** | 1 | **0.2** |
| 3 | 5 | -17 | 0 | **1.6** | 0 | **0.0** | 34 | 24 | **68.4** | 1 | **2.4** |
|  | 6 | -72 | -44 | **61.7** | -10 | **13.7** | 446 | 404 | **90.5** | 365 | **81.8** |
| 4 | 7 | 0 | 0 | **100.0** | 0 | **0.0** | 221 | 159 | **72.0** | 10 | **4.4** |
|  | 8 | -39 | -29 | **75.4** | 0 | **0.0** | 410 | 283 | **69.0** | 4 | **1.1** |
|  | 9 | -14 | -8 | **59.9** | -3 | **19.4** | 36 | 0 | **1.2** | 0 | **0.0** |
| 5 | 10 | -158 | -70 | **44.3** | -66 | **41.5** | 329 | 278 | **84.4** | 165 | **50.2** |
|  | 11 | -67 | -5 | **7.1** | -4 | **5.4** | 61 | 13 | **20.8** | 0 | **0.0** |
|  | 12 | -1315 | -1315 | **100.0** | -1279 | **97.2** | 61 | 23 | **37.3** | 0 | **0.0** |
| 6 | 13 | -552 | -465 | **84.2** | -219 | **39.7** | 1595 | 1364 | **85.5** | 0 | **0.0** |
|  | 14 | -710 | -466 | **65.7** | -403 | **56.8** | 424 | 373 | **88.1** | 0 | **0.0** |
| 7 | 15 | -232 | -120 | **51.8** | -118 | **51.0** | 142 | 95 | **67.2** | 57 | **39.9** |
| 8 | 16 | -609 | -440 | **72.4** | -367 | **60.3** | 83 | 41 | **49.0** | 5 | **6.5** |
|  | 17 | -227 | -134 | **58.9** | -63 | **27.5** | 229 | 161 | **70.4** | 23 | **9.9** |

**Sources**: This table is based on BLS Employment Projections 2019-2029, Table 1.2 “Employment by detailed occupation, 2019 and projected 2029” and Table 1.12 “Factors affecting occupational utilization, projected 2019–29”. Occupations are clustered using Ward hierarchical method based on 72 principal components derived from 220 occupational descriptors from O*NET. Own calculations.
